# Ferroptosis and aging: Inducing and catalyzing neurodegenerative diseases

**DOI:** 10.4103/NRR.NRR-D-25-00710

**Published:** 2025-09-03

**Authors:** Qifeng Song, Shi Sun, Yuxiu Song, Yashi Wang, Yin Yuan, Lixin Zhang, Qian Cui

**Affiliations:** 1 Department of Rehabilitation, Shengjing Hospital of China Medical University, Shenyang, Liaoning Province, China; 2 Department of Critical Care Medicine, Shengjing Hospital of China Medical University, Shenyang, Liaoning Province, China

**Keywords:** aging, Alzheimer’s disease, exosomes, ferroptosis, iron overload, lipid peroxidation, neural regeneration, Parkinson’s disease, physical therapy, stem cells

## Abstract

Ferroptosis is a newly recognized form of programmed cell death characterized by iron overload-dependent lipid peroxidation. These pathological phenomena are often observed in neurodegenerative diseases. Aging is an irreversible process characterized by the deterioration of tissue and cell function. It has been shown to contribute to neurodegenerative diseases and increase susceptibility to ferroptosis. Therefore, ferroptosis may be involved in the progression of neurodegenerative diseases as a pathogenic factor, and aging is the common catalyst of both processes. The purpose of this review is to elucidate the latest progress on the mechanisms related to ferroptosis in neurodegenerative diseases, including iron overload, lipid peroxidation, antioxidant defense, cell membrane repair, and the regulation of autophagy and transcription factors. We also explored the relationship between ferroptosis and aging and reported that aging can induce ferroptosis by increasing iron overload, enhancing lipid peroxidation, and exacerbating autophagy disorders. Since ferroptosis is a pathogenic factor in neurodegenerative diseases, we screened gene bank databases and found that many genes associated with ferroptosis and neurodegenerative diseases overlap. Additionally, genes related to both the peroxidation pathway and ferroptosis are enriched. Ferroptosis occurs under conditions of age-related iron accumulation and lipid enrichment, as well as due to disorders in autophagy levels and transcription factors. Furthermore, in various neurodegenerative diseases, specific pathological changes or products can also contribute to the occurrence of ferroptosis. Finally, based on animal studies and clinical trials involving ferroptosis inhibitors, physical therapies, stem cell treatments, and exosome therapies in neurodegenerative diseases, it has been found that inhibiting ferroptosis can effectively reverse neurological dysfunction and cognitive impairment associated with these conditions. However, given various limitations, the conclusions of some animal studies and clinical trials have not been ideal, indicating that further large-scale research is necessary. Taken together, ferroptosis induces aging-related neurodegenerative diseases and neuronal cell death, triggering disease onset and progression. Ferroptosis inhibitors, physical therapies, stem cell treatments, and exosome therapies show great potential for inhibiting ferroptosis in neurodegenerative disease.

## Introduction

Various types of cell death, as fundamental physiological processes, play a vital role throughout an organism’s life cycle, from initial embryonic development to the maintenance of homeostasis and ultimately aging and death (Bertheloot et al., 2021). In addition to their physiological roles, cell death processes can also have pathological implications, as abnormal cell death often leads to disease (Yan et al., 2021). Cell death can be categorized into two main types: accidental cell death and regulated cell death (RCD). Accidental cell death is primarily an uncontrolled process that occurs in response to external stimuli or injuries, whereas RCD is a type of cell death that is regulated by biological factors and plays a more extensive role in the life cycle of organisms (Tong et al., 2022). Ferroptosis is a specific mode of RCD characterized by disruptions in iron metabolism and the accumulation of lipid peroxides. Numerous studies have confirmed that ferroptosis is crucial for sustaining human health and is involved in the occurrence and progression of various diseases (Jiang et al., 2021; Sun et al., 2023).

Neurodegenerative diseases, including Alzheimer’s disease (AD), Parkinson’s disease (PD), Huntington’s disease (HD), and amyotrophic lateral sclerosis (ALS), have an incidence that is positively correlated with aging. Currently, there is no cure for these diseases, which places a heavy burden on society and patients’ families. It is estimated that by 2050, neurodegenerative diseases will affect approximately 150 million people worldwide, resulting in economic burdens of up to 10 trillion US dollars (Temple, 2023). One of the main causes of neurodegenerative diseases is the premature death of neural cells that are essential for maintaining the function of the nervous system (Yuan and Ofengeim, 2024). The abnormal activation of RCD in neural cells is a characteristic pathological change observed in these diseases (Moujalled et al., 2021). Furthermore, numerous studies have confirmed that iron deposition (Ayton et al., 2020; Ma et al., 2021) and lipid peroxidation (Ansari and Scheff, 2010; Hatami et al., 2018) are significant risk factors for neurodegenerative diseases. Ferroptosis inhibitors appear to delay disease progression (Yan et al., 2021). It is well known that aging-related iron deposition and lipid peroxidation also contribute to ferroptosis (Coradduzza et al., 2023). Additionally, we have previously elucidated that ferroptosis is a significant mode of cell death following spinal cord injury (Song et al., 2024), and inhibiting ferroptosis shows promise for reversing neurological dysfunction (Song et al., 2023a). Therefore, ferroptosis is likely to be a key contributor to the pathogenesis of neurodegenerative diseases and is expected to emerge as a new therapeutic target.

**Figure 1:**
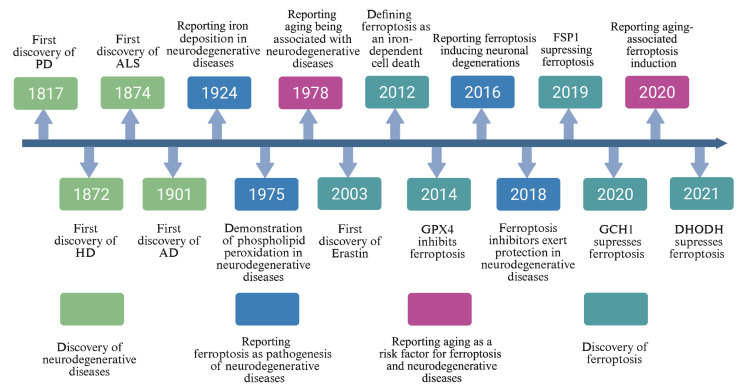
Timeline of ferroptosis and aging-related neurodegenerative diseases. A timeline illustrating the development of neurodegenerative diseases and ferroptosis, as well as the evolving relationship between aging, ferroptosis, and neurodegenerative diseases. Created with BioRender.com. AD: Alzheimer’s disease; ALS: amyotrophic lateral sclerosis; DHODH: dihydroorotate dehydrogenase; FSP1: ferroptosis suppressor protein 1; GCH1: guanosine triphosphate cyclohydrolase 1; GPX4: glutathione peroxidase 4; HD: Huntington’s disease; PD: Parkinson’s disease.

In this review, we summarize the latest advancements in research regarding the mechanisms and pathways of ferroptosis, its relationship with aging, its association with neurodegenerative diseases, and potential treatments (**Figure 1**). Based on the findings of relevant studies, we conclude that the mechanism of ferroptosis is complex and regulated by multiple factors. Numerous studies have confirmed that ferroptosis can contribute to the pathogenesis of neurodegenerative diseases through various mechanisms, mediating neuronal cell death. Additionally, ferroptosis can trigger the onset of these diseases and accelerate their progression.

Aging serves as a common risk factor for both ferroptosis and neurodegenerative diseases, with their incidence significantly increasing as individuals age. Inhibiting ferroptosis has been shown to effectively reverse neurological dysfunction and cognitive impairment associated with neurodegenerative diseases. Ferroptosis inhibitors, along with physiotherapy, stem cell therapy, and exosome therapy, exhibit clinical feasibility and promising potential for reducing ferroptosis in these conditions. The research described in this review provides evidence for neural regeneration in neurodegenerative diseases and opens new avenues for improving outcomes by targeting ferroptosis. This represents a significant step toward reversing the prognosis of neurodegenerative diseases.

In summary, downregulating ferroptosis in neural cells is expected to delay the onset and progression of aging-related neurodegenerative diseases while offering new directions and targets for treatment. However, there are very few direct reviews addressing ferroptosis as a mechanism in the pathogenesis of aging-related neurodegenerative diseases. In this review, we provide a theoretical foundation and personal insights into the role of ferroptosis in neurodegeneration and the corresponding therapies. Our aim is to establish a basis for further research, identify new targets for neural regeneration, and encourage greater attention from professionals in this field to conduct a broader range of in-depth studies. Ultimately, we hope to provide a new direction for the treatment of neurodegenerative diseases.

## Search Strategy

All the authors conducted a comprehensive search of the PubMed and Web of Science databases using targeted keywords, including “Neurodegenerative diseases,” “Ferroptosis,” “Aging,” “Alzheimer’s disease,” “Parkinson’s disease,” “Huntington’s disease,” “Amyotrophic lateral sclerosis,” “Iron,” “Lipid,” “Ferroptosis inhibitor,” “Physiotherapy,” “Stem cell,” and “Exosome.” Various combinations of these keywords were used to retrieve articles and reviews, and the titles and abstracts of all identified articles and reviews were screened. The screening focused primarily on the relationships among aging, ferroptosis, and aging-related neurodegenerative diseases, as well as the role of ferroptosis in these diseases and relevant therapies. Although a total of 357 references were included to ensure a comprehensive understanding from 2006 to 2025, we prioritized recent studies published between 2020 and 2025. However, key earlier studies and reviews of significant relevance were also included to provide a well-rounded perspective in our review.

All 357 selected references met the following selection criteria. First, the primary content of all the references is highly relevant to the subject of this review. Of these, 259 (72.5%) are directly related to the relationship among ferroptosis, aging, and neurodegenerative diseases, while the remaining 98 (27.5%) primarily address the pathological changes and epidemiological characteristics of neurodegenerative diseases, as well as other forms of cell death. Second, all references were published in reputable Science Citation Index journals with impact factors. Finally, most of the references are from literature published in the past 5 years, and those with similar research contents or conclusions are relatively recent.

In addition, the research in the references involves a wide range of animal models, including rats, mice, zebrafish, and *Drosophila*. Moreover, the same animal model may exhibit certain differences depending on the specific diseases or experimental requirements. Given the wide variety and complexity of the mechanisms related to ferroptosis and neurodegenerative diseases, a large number of genes or proteins were involved in this review, including, but not limited to: amyloid-β (Aβ), acyl-CoA synthetase long-chain family member 4 (ACSL4), amyloid precursor protein (APP), α-synuclein (α-syn), dihydroorotate dehydrogenase (DHODH), divalent metal transporter 1 (DMT1), ferroptosis suppressor protein 1 (FSP1), ferroportin 1 (FPN1), guanosine triphosphate (GTP) cyclohydrolase-1 (GCH1), glutathione peroxidase 4 (GPX4), nuclear factor erythroid 2-related factor 2 (Nrf2), solute carrier family 7 member 11 (SLC7A11), and transferrin (TF). These genes can regulate ferroptosis or neurodegenerative diseases through different pathways and are associated with ferroptosis and neurodegenerative diseases in their respective ways. Additionally, based on their mechanism of action, the aforementioned genes can be divided into two categories: those that activate ferroptosis to induce or exacerbate neurodegenerative diseases, and those that inhibit ferroptosis to delay the onset of neurodegenerative diseases or improve disease prognosis.

Furthermore, to identify the overlapping genes between ferroptosis and neurodegenerative diseases and to explore the closely related genes and pathways, we searched the GeneCards database for genes associated with aging-related neurodegenerative diseases, including AD, PD, HD, ALS, and ferroptosis. We used Boolean models to find matching entries and then used a practical scoring function to calculate the correlation and rank the results, forming respective gene banks. The gene banks were compared to identify overlapping genes. Subsequently, the overlapping genes between all the diseases and ferroptosis were imported into the SRRING database, and functional network enrichment analysis was performed. This analysis provided insight into the genes in the biological process category of the Gene Ontology database and the pathways in the KEGG database. These genes and pathways exhibited relatively close associations and high strength rankings.

## Ferroptosis

Ferroptosis was first defined in 2012. Dixon et al. (2012) reported a novel mode of non-apoptotic cell death characterized by iron metabolism disorder and the accumulation of lipid reactive oxygen species (ROS) during selective lethality in oncogenic rat sarcoma virus oncogene (RAS) mutant cell lines treated with erastin; this mode was ultimately referred to as ferroptosis. The morphological characteristics of ferroptosis include a decrease in mitochondrial volume, a significant increase in membrane density, a decrease in the number or disappearance of cristae, and rupture of the outer membrane, while the nucleus remains intact (Li et al., 2020). After ferroptosis, cells undergo rupture of the plasma membrane mediated by pore formation and spread through cell populations in a wave-like manner. Cell-to-cell transmission is also a characteristic of ferroptosis, although the specific mechanism remains unclear (Riegman et al., 2020). Various organelles in cells are involved in ferroptosis and play different roles. The plasma membrane is a key site of lipid peroxidation and an important component of the antioxidant defense system. Thus, the executive center of ferroptosis is the plasma membrane. Meanwhile, regulatory activities of ferroptosis occur mainly in the cytoplasm. Other organelles, including the nucleus, mitochondria, endoplasmic reticulum (ER), Golgi apparatus, and lysosome, are involved in a variety of critical processes. These include transcription, ROS synthesis, phospholipid synthesis and remodeling, iron metabolism and release, lipid metabolism, and the regulation of lipid peroxidation (Dixon and Olzmann, 2024). Each of these functions is essential for the occurrence and progression of ferroptosis.

In contrast to previously confirmed modes of cell death (**Additional Table 1**), ferroptosis is characterized by its unique mechanism of iron-dependent lipid peroxidation and distinct mitochondrial features. Therefore, we aimed to explore the specific role of ferroptosis in the occurrence and progression of diseases by comparing it with various other cell death modes. We believe that, unlike previous cell death mechanisms, ferroptosis may offer new insights into the pathogenesis of neurodegenerative diseases, particularly in relation to iron metabolism, lipid peroxidation, and mitochondrial changes. This understanding may also provide new directions for treatment.

**Additional Table 1:** Various forms of cell death

Cell death	Cellular morphological changes	Characteristic mechanisms	Major regulators	Others
Necrosis	Nuclear pyknosis, nuclear fragmentation, karyolysis, cytoplasmic coagulation or dissolution, cell swelling, plasma membrane rupture, and cell content outflow	Stress, injury, and poisoning	Uncontrolled	Passive cell death, and no energy consumption
Apoptosis	Cell shrinkage, membrane blebbing, chromosome condensation, and formation of apoptotic bodies	Activation of caspase	Initiator caspase, effector caspase, and BCL2 family	Divided into external, internal and endoplasmic reticulum stress pathways
Autophagy	Successive formation of phagophores, autophagosomes, and autolysosomes	Increased autophagy flux, lysosomal activity, and LC3-I to LC3-II conversion	Na^+^/K^+^-ATPase, mTOR, BECN1,and AMPK	ADCD and AMCD
Pyroptosis	Cell swelling (weaker than necrosis), formation of pyroptotic bodies, membrane pore-mediated plasma membrane rupture, and inflammatory response	Activation of gasdermin, gasdermin cleavage	Gasdermin C/D/E, caspase-1/3/4/5/8/11	Related to inflammation, including canonical, non-canonical, apoptotic caspases-mediated pyroptosis and granzyme-based pathway
Necroptosis	Cell swelling, rupture of plasma membrane, and cell contents outflow	Activation of RIPK1, RIPK3 and MLKL, and necrosome formation	RIPK1, RIPK3, and MLKL	Backup measures for apoptosis, depending on the activity of caspase-8
Ferroptosis	Reduced mitochondrial volume, thickened membranes, and reduced cristae, rupture of mitochondrial membrane, and nucleus remains intact	Iron overload, lipid peroxidation, and thiol disorder	GPX4, FSP1, DHODH, GCH1, and ACSL4	Iron dependent, characteristic changes in mitochondria
Cuproptosis	Mitochondrial shrinkage, plasma membrane rupture, endoplasmic reticulum injury, and chromatin damage	Copper overload, mitochondrial lipoylated proteins, Fe-S cluster protein loss	FDX 1, SLC31A1, and ATP7B	Copper dependent

This table represents cellular morphological manifestations, characteristic mechanisms, and major regulators of various forms of cell death. ACSL4: Acyl-CoA synthetase long-chain family member 4; ADCD: autophagy-dependent cell death; AMCD: autophagy-mediated cell death; AMPK: adenosine 5'-monophosphate (AMP)-activated protein kinase; ATP7B: ATPase copper-transporting beta; BCL2: B-cell lymphoma 2; BECN1: Beclin 1; DHODH: dihydroorotate dehydrogenase; FDX1: ferredoxin 1; FSP1: ferroptosis suppressor protein 1; GCH1: guanosine triphosphate cyclohydrolase 1; GPX4: glutathione peroxidase 4; LC3:microtubule-associated protein light chain 3; MLKL: mixed lineage kinase domain-like protein; mTOR: mammalian target of rapamycin; RIPK: receptor interacting protein kinase; SLC31A1: solute carrier family 31 member A1.

As mentioned above, iron overload and lipid peroxidation are the main biochemical characteristics of ferroptosis (Chen et al., 2021a) and are also essential conditions for its occurrence (Riegman et al., 2020). Additionally, the anti-lipid peroxidation system, cell membrane repair system, autophagy, transcription factors, and genetic regulation also influence the occurrence and development of ferroptosis (Tang et al., 2021).

### Iron metabolism

#### Physiological iron metabolism

Under physiological conditions, the uptake, utilization, storage, and metabolism of iron is a complex process that involves key proteins and receptors, including DMT1, FPN1, TF, transferrin receptor 1 (TfR1), ferritin, and hepcidin (Zhang et al., 2022).

At the cellular level, iron ions typically enter the cell as trivalent iron, bound to TF and its receptor, transferrin receptor 1 (TfR1). This trivalent iron is then reduced to ferrous iron by the metal reductase STEAP (six-transmembrane epithelial antigen of prostate) family and released into the cytoplasm through the activity of DMT1 (Koleini et al., 2021). In the cytoplasm, intracellular free ferrous iron is added to the labile iron pool (LIP) and is subsequently utilized or stored in ferritin during cellular metabolism (Sukhbaatar and Weichhart, 2018). Additionally, cellular iron homeostasis is primarily maintained by iron regulatory proteins (IRPs), which regulate the transcription of iron-related proteins by binding to the iron-responsive element (IRE) region in the untranslated regions (UTR) of their mRNAs (Zhang et al., 2022).

#### Iron overload and ferroptosis

**Figure 2:**
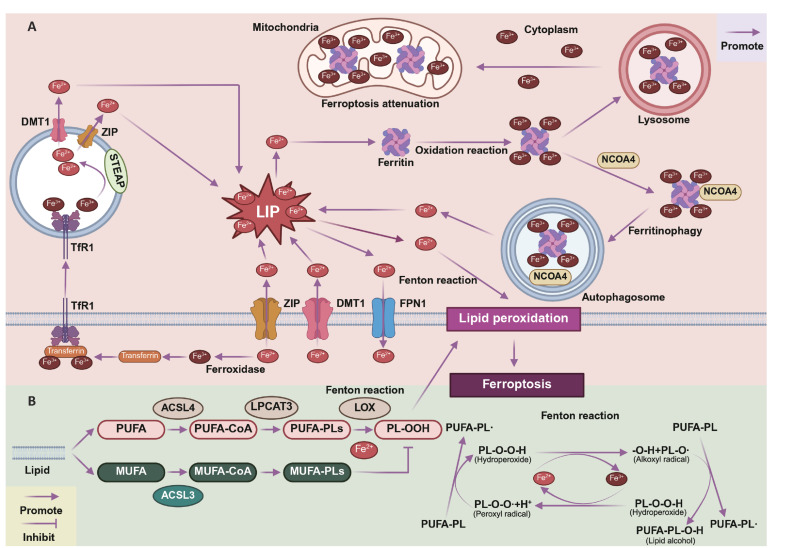
Iron metabolism and lipid peroxidation in ferroptosis. (A) Iron metabolism in ferroptosis. An increase in iron input, a decrease in iron output, and increased ferritinophagy collectively lead to iron overload in the intracellular labile iron pool. This overloaded iron undergoes the Fenton reaction, ultimately resulting in ferroptosis. (B) Lipid peroxidation in ferroptosis. PUFAs/MUFAs are converted into PUFA-PLs/MUFA-PLs through a series of reactions. PUFA-PLs undergo the Fenton reaction catalyzed by overloaded iron and LOX, producing lipid peroxidation products that mediate ferroptosis. In contrast, MUFA-PLs can inhibit ferroptosis. Created with BioRender.com. ACSL3: Acyl-CoA synthetase long-chain family member 3; ACSL4: acyl-CoA synthetase long-chain family member 4; DMT1: divalent metal transporter 1; FPN1: ferroportin 1; LIP: labile iron pool; LOX: lipoxygenase; LPCAT3: lysophosphatidylcholine acyl-transferase 3; MUFA: monounsaturated fatty acid; NCOA4: nuclear receptor coactivator 4; PLOOH: phospholipid hydroperoxide; PUFA: polyunsaturated fatty acid; PUFA-PLs: polyunsaturated fatty acid-containing phospholipids; STEAP: six-transmembrane epithelial antigen of prostate; TfR1: transferrin receptor 1; ZIP: ZRT/IRT-like protein.

Under special conditions such as iron import, hemoglobin degradation, and ferritinophagy, the LIP becomes overloaded (Sawicki et al., 2023). Furthermore, the abnormal downregulation of FPN1, the only known protein capable of transporting unstable ferrous iron to the extracellular space, also contributes to LIP overload (Fang et al., 2023). In addition to directly causing oxidative damage, unstable free ferrous iron participates in the synthesis of lipoxygenase (LOX), a key enzyme in lipid peroxidation. LOX mediates lipid peroxidation together with unstable ferrous iron after free radicals are generated through the Fenton reaction, ultimately inducing ferroptosis (Sun et al., 2022b; **Figure 2A**).

### Lipid peroxidation

Lipids are the primary components of cell membranes and play crucial roles in maintaining cell structure and function. As mentioned above, lipid peroxidation mediated by iron overload is at the core of ferroptosis in cells. Lipid peroxidation begins when free radicals or other oxidants attack the carbon double bonds of lipids. The main substrate for lipid peroxidation is polyunsaturated fatty acids (PUFAs) (Rochette et al., 2022), which undergo sequential actions via ACSL4 and lysophosphatidylcholine acyltransferase 3 (LPCAT3) to form PUFA-containing phospholipids (PUFA-PLs). An increased content of PUFA-PLs in the cell membrane heightens the intrinsic sensitivity of cells to ferroptosis, particularly those containing a bisallylic group (−CH=CH−CH2−CH=CH−) (Liang et al., 2022). The PUFA-PLs that undergo peroxidation generate phospholipid hydroperoxides (PLOOH; **Figure 2B**). In the context of unstable iron overload, PLOOH-associated lipid radicals further propagate the peroxidation reaction to adjacent PUFA-PLs, generating new PLOOH. These reactions continue until the massive accumulation of PLOOH leads to cell membrane rupture and cell death, or until the cell survives due to the neutralization of the oxidative reaction or depletion of the substrate (Liang et al., 2022; Wang et al., 2023a). In addition to producing PLOOH, lipid peroxidation also results in the formation of various toxic coproducts, including malondialdehyde (MDA), propionaldehyde, hexanal, and 4-hydroxynonenal (4-HNE).

### Anti-lipid peroxidation system

Under physiological conditions, cells have an inhibitory self-protection mechanism against lipid peroxidation. The cystine/glutamate transporter system (Xc-system) is an important member of the amino acid transporter family, consisting of the light chain SLC7A11 and the heavy chain SLC3A2 (solute carrier family 3 member 2). SLC7A11 serves as a crucial functional unit of the Xc-system, demonstrating high specificity for cystine and glutamate. Its functions include facilitating cystine uptake and glutamate release, promoting the synthesis of glutathione (GSH), and preventing lipid peroxidation in cells. Meanwhile, SLC3A2 acts as the chaperone protein for SLC7A11, maintaining its stability and regulating its transport.

#### Elimination of phospholipid peroxidades

GPX4 was first shown to effectively clear intracellular PLOOH (Zhang et al., 2024a). GSH, a strong antioxidant that functions as both a cofactor and a downstream factor of GPX4, is also involved in the clearance of PLOOH (Chen et al., 2021b). Therefore, thiols, represented by GSH, are considered the third pillar of ferroptosis, alongside iron overload and lipid peroxidation (Bayır et al., 2023). The GPX4-GSH pathway is regarded as the classic inhibitory pathway for ferroptosis and was the only pathway identified for a period (Bersuker et al., 2019).

#### Producing antioxidants

A follow-up study by Doll et al. (2019) confirmed that FSP1 inhibits ferroptosis by reducing ubiquinone in parallel with the canonical GPX4 pathway. Moreover, DHODH was shown to inhibit ferroptosis in the inner mitochondrial membrane by reducing ubiquinone to ubiquinol, thereby converting it into a free radical-trapping antioxidant (RTA) with antiferroptotic activity (Mao et al., 2021). However, a recent study revealed that DHODH inhibitors enhance the sensitivity of cancer cells to ferroptosis by inhibiting FSP1 rather than DHODH (Mishima et al., 2023). Therefore, we believe that DHODH plays a role in ferroptosis, despite its seemingly minor effects. By performing a genome-wide activation screen, researchers subsequently determined that GCH1 and its metabolite tetrahydrobiopterin/dihydrobiopterin (BH4/BH2) inhibit ferroptosis by exerting lipid remodeling and antioxidant effects, respectively (Kraft et al., 2020).

#### Remodeling the cellular phospholipid structure

**Figure 3:**
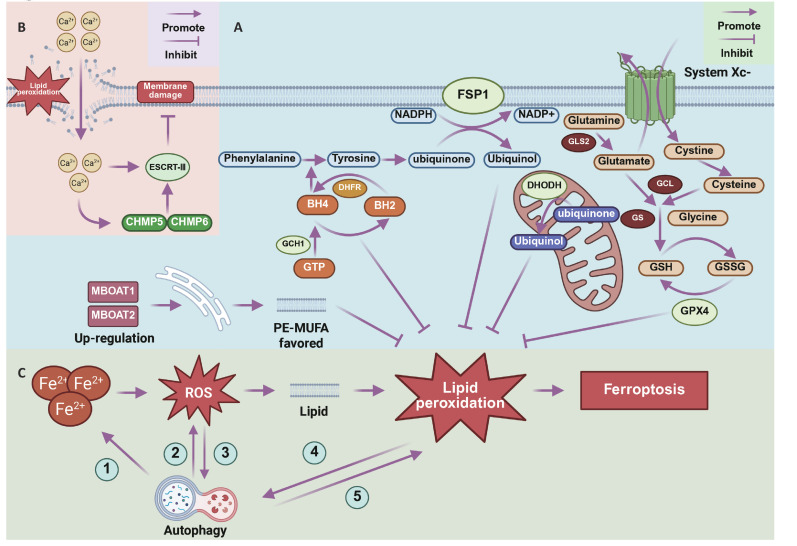
Regulation of ferroptosis by anti-lipid peroxidation systems, membrane repair mechanisms, and autophagy. (A) Lipid peroxidation defence systems in ferroptosis. (B) Membrane repair system in ferroptosis. After lipid peroxidation, calcium influx activates the ESCRT- III complex near the site of membrane damage, ultimately exerting membrane repair effects. (C) Autophagy regulates ferroptosis. ① Ferritinophagy and excessive mitophagy. ② Autophagic proteins promote iron metabolism and lipid peroxidation, amplifying ROS generation in ferroptosis; autophagy and the ROS feedback loop. ③ Direct oxidation of autophagy related proteins; regulation of autophagy regulatory factors; regulation of transcription factors and effect on autophagy related gene expression; activation of mitophagy. ④ Generation of ROS; damage to lipid droplets indirectly promotes autophagy; inducing autophagy to clear lipid peroxidation products. ⑤ Lipophagy; CMA; clockophagy; macrolipophagy. Created with BioRender.com. BH2: Dihydrobiopterin; BH4: tetrahydrobiopterin; CHMP: charged multivesicular body protein; CMA: chaperon-mediated autophagy; DHFR: dihydrofolate reductase; DHODH: dihydroorotate dehydrogenase; ESCRT-III: endosomal sorting complex required for transport-III; FSP1: ferroptosis suppressor protein 1; GCH1: guanosine triphosphatecyclohydrolase 1; GCL: glutamate cysteine ligase; GLS2: glutamine synthase 2; GS: glutathione synthase; GSH: Glutathione; GSSG: glutathione disulfide; GTP: guanosine triphosphate; GPX4: glutathione peroxidase 4; MBOAT: membrane-bound O-acyltransferase; NADPH: nicotinamide adenine dinucleotide phosphate; PE-MUFA: phosphatidylethanolamine polyunsaturated fatty acid; ROS: reactive oxygen species.

A recent genome-wide screen revealed that the phospholipid-modifying enzymes membrane-bound O-acyltransferase (MBOAT) 1 and 2 can inhibit ferroptosis by remodeling the cellular phospholipid structure, and the mechanism may involve reducing lipid peroxidation substrates (Liang et al., 2023a). To date, the anti-lipid peroxidation pathways associated with ferroptosis can be divided into three main categories: (1) pathways mediated by GPX4 to eliminate phospholipid peroxides; (2) pathways mediated by FSP1, DHODH, and GCH1 to produce antioxidants; and (3) pathways mediated by MBOAT1 and 2, which reduce ferroptosis reaction substrates by remodeling the cellular phospholipid structure (Liu et al., 2023c; **Figure 3A**).

### Cell membrane repair system

In addition to limiting lipid peroxidation of the plasma membrane through the antioxidant systems mentioned above, cells can initiate plasma membrane repair through a series of processes, including Ca^2+^ influx, vesicle formation, exocytosis, and endocytosis (Andrews et al., 2014). Following lipid peroxidation, the continuous increase in cytosolic Ca^2+^ concentration is regulated by osmotic pressure and triggered by nanoscale pores in the plasma membrane. This increase is an early sign of ferroptosis, occurring before the complete rupture of cells, and induces the activation of a membrane repair mechanism dependent on the endothelial sorting complex required for transport-III (ESCRT-III). ESCRT-III-dependent membrane repair is considered a delayed cytoprotective mechanism that antagonizes ferroptosis in cells (Pedrera et al., 2021). The elevation of cytosolic Ca^2+^ concentration is key to activating the ESCRT-III complex. A sustained increase in cytosolic Ca^2+^ triggers the activation and recruitment of the ESCRT-III complex to the cell membrane. The ESCRT-III complex is composed of charged multivesicular body proteins (CHMPs), which tend to aggregate near the injury site of the cell membrane to facilitate repair. Among the various CHMPs, CHMP5 and CHMP6 are core components of the ESCRT-III complex (Dai et al., 2020b; **Figure 3B**). Additionally, FSP1, mentioned in the previous section, has been shown to play a role in ESCRT-III-dependent membrane repair; FSP1 knockdown inhibits the aggregation of CHMP5 and CHMP6 in the plasma membrane of ferroptotic cells (Dai et al., 2020c).

### Autophagy and ferroptosis

#### Autophagy regulates ferroptosis

Ferroptosis was initially shown to be independent of previously discovered modes of cell death (Dixon et al., 2012). Early studies demonstrated that moderate levels of autophagy are beneficial for maintaining cell homeostasis (Chen et al., 2019; Liu et al., 2023b). However, subsequent studies have indicated that excessive levels of autophagy can promote ferroptosis under certain conditions (Zhou et al., 2020a; Chu et al., 2023b). Autophagy can be divided into selective and nonselective processes, both of which can regulate ferroptosis (Lv et al., 2023b). Studies have shown that appropriate levels of selective autophagy can achieve a protective effect by inhibiting ferroptosis through the selective removal of damaged or dysfunctional cellular components via mechanisms such as reticulophagy (ER-phagy) (Liu et al., 2022c), lysophagy (Li et al., 2023b), and chaperone-mediated autophagy (CMA)-mediated ACSL4 degradation (Liu et al., 2022a). The selective autophagy mechanisms that activate ferroptosis can be categorized into two main categories: regulation of iron levels and regulation of lipid peroxidation. The regulation of iron levels primarily includes ferritinophagy (Gao et al., 2016), such as ROS-mediated autophagy leading to intracellular iron overload by degrading ferritin and inducing the expression of TfR1 (Park and Chung, 2019). The regulation of lipid peroxidation involves mechanisms such as lipophagy (Bai et al., 2019), clock autophagy (Yang et al., 2019), CMA (Wu et al., 2019), and mitophagy (Bi et al., 2024; **Figure 3C**). The former two mechanisms mainly involve the uptake, storage, and utilization of lipids, while the latter two primarily regulate the anti-lipid peroxidation system and mitochondrial function. Nonselective autophagy mainly regulates ferroptosis by affecting the iron export regulatory network and regulating the biosynthesis of PUFAs (Lv et al., 2023a).

Autophagy can also regulate multiple pathways simultaneously to induce ferroptosis. Recent findings have confirmed that autophagy mediated by transmembrane protein 164 (TMEM164) can lead to the degradation of ferritin, lipid droplets, and GPX4, thereby inducing ferroptosis (Liu et al., 2023a). Moreover, subsequent studies have confirmed that there are still numerous novel mechanisms by which autophagy regulates ferroptosis (Zhang et al., 2020b; Chen et al., 2023, 2024b).

#### Ferroptosis regulates autophagy

On one hand, ferroptosis can be divided into two categories: autophagy-dependent ferroptosis, in which an autophagy mechanism is required to induce ferroptosis, and autophagy-independent ferroptosis, in which autophagy occurs alongside ferroptosis but does not directly induce it and may even play a protective role during ferroptosis. On the other hand, ferroptosis can also regulate autophagy. Oxidative reactions that occur after ferroptosis can lead to autophagy. Additionally, the lipid peroxidation reaction and its products following ferroptosis can catalyze autophagy. Although direct evidence that ferroptosis regulates autophagy is limited, studies have supported that ferroptosis can regulate autophagy through different pathways (Lv et al., 2023a).

#### Relationships between ferroptosis and autophagy

In summary, the association between ferroptosis and autophagy is bidirectional, and the specific dynamics depend on the cell type and environment. The precise mechanisms remain unclear (Wang et al., 2023a). An analysis of the studies mentioned above revealed that disordered autophagy may induce ferroptosis by degrading antioxidant proteins, particularly GPX4 and SLC7A11 (Chen et al., 2021b; Liu et al., 2023a). Additionally, a reduction in SLC7A11 affects cysteine levels, impacting not only the synthesis of GSH but also driving autophagy activation through amino acid starvation signals and peroxidation reactions, thereby exacerbating ferroptosis through processes such as ferritinophagy. In contrast, under specific conditions, autophagy may support the synthesis of the antioxidant system by recycling metabolites such as amino acids and fatty acids, thereby indirectly inhibiting ferroptosis.

The specific roles that autophagy plays in ferroptosis at different stages are also controversial. For example, after iron overload leads to the inactivation of GPX4, autophagy may be activated to delay the onset of ferroptosis, or it may exacerbate ferroptosis. Defining the role of autophagy in its cross-regulation with ferroptosis warrants careful consideration.

### Transcription factors and genetic regulation

#### Transcription factors and ferroptosis

Transcription factors play a crucial role in the process of ferroptosis by regulating gene expression through the recognition of specific DNA sequences. Consequently, the role of transcription factors in ferroptosis is primarily determined by the genes they regulate and the diverse environments in which they operate (Dai et al., 2020a). The transcription factors involved in the regulation of ferroptosis include Nrf2, tumor protein 53 (TP53), and ATF. Nrf2 exerts inhibitory effects on ferroptosis by interfering with iron metabolism, lipid peroxidation, and the antioxidant system through the regulation of various genes that negatively regulate ferroptosis, such as ferritin heavy chain 1 (FTH1), FPN1, peroxisome proliferator-activated receptor gamma (PPARG), glucose-6-phosphate dehydrogenase (G6PD), SLC7A11, and GPX4 (Dodson et al., 2019; Anandhan et al., 2020, 2023). In contrast to Nrf2, TP53 plays a dual role in ferroptosis (Kang et al., 2019). On one hand, TP53 increases ferroptosis by transcriptionally repressing SLC7A11 expression (Yao et al., 2024a) or promoting spermidine/spermine N1-acetyltransferase 1 (SAT1) (Ou et al., 2016). On the other hand, TP53 can inhibit ferroptosis by transcriptionally inhibiting dipeptidyl peptidase-4 (DPP4) activity (Xie et al., 2017) or promoting the expression of cyclin-dependent kinase inhibitor 1A (CDKN1A/p21) (Tarangelo et al., 2018). Finally, ATFs, including ATF3 and ATF4, are regulated by ER stress. ATF3 has dual effects on regulating ferroptosis by activating the transcription of downstream genes (Liu et al., 2024c). On one hand, ATF3 inhibits ferroptosis by upregulating GPX4 expression, thereby reducing Fe2+ accumulation, ROS production, and MDA release (Liu et al., 2022b). On the other hand, ATF3 can also negatively regulate SLC7A11 expression, leading to a decrease in GSH and the activation of ferroptosis (Wang et al., 2020b). ATF4 also plays dual roles in the inhibition and activation of ferroptosis through multiple pathways (Tang et al., 2024).

#### Epigenetic factors and ferroptosis

In addition to transcription factors, epigenetic factors, including DNA modifications, non-coding RNAs, and histone modifications, can affect ferroptosis (Lan et al., 2023). Non-coding RNAs mainly include microRNAs (miRNAs), circular RNAs (circRNAs), and long non-coding RNAs (lncRNAs) (Yan and Bu, 2021). Epigenetic factors play a dual role in ferroptosis through complex and variable mechanisms, with this complexity and variability arising from the different pathways and downstream targets they regulate (Yang et al., 2023b).

In recent years, research on the epigenetic regulation of ferroptosis, particularly involving non-coding RNAs, has gained popularity (Wang et al., 2024b). The study by Lu et al. (2022) demonstrated that miR-23a-3p inhibits ferroptosis by regulating ACSL4 to reduce iron overload and lipid peroxidation. Similarly, miR-125b-5p can inhibit ferroptosis through the Kelch-like ECH-associated protein 1 (Keap1)/Nrf2/GPX4 pathway (Shen et al., 2023). In contrast, miR-30b-5p activates ferroptosis by negatively regulating the SLC7A11/GSH and paired box 3 (Pax3)/FPN1 pathways (Zhang et al., 2020a).

## Ferroptosis and Aging

**Figure 4:**
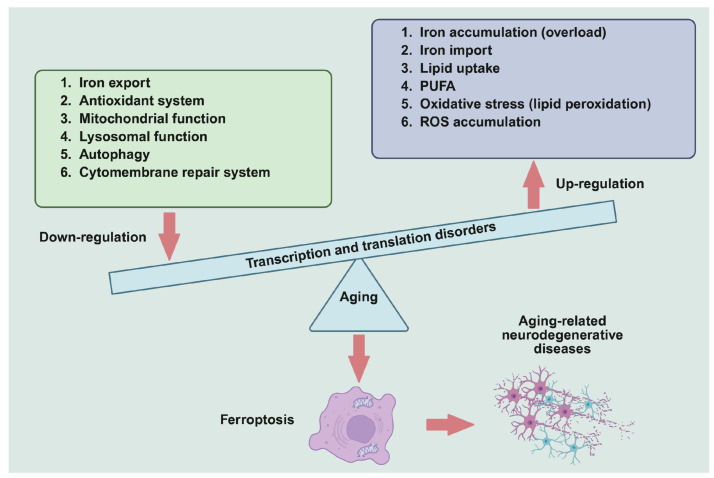
Aging and ferroptosis. After aging, neurons undergo iron deposition, increased lipid uptake and synthesis, dysfunction of the antioxidant system, heightened oxidative stress response, downregulation of autophagy, dysfunction of the cytomembrane repair system, and disorders in transcription and translation. All of these changes considerably lower the threshold for the occurrence of ferroptosis, ultimately leading to aging-related neurodegenerative diseases.

Aging is a complex, irreversible process characterized by signs such as genomic instability, telomere depletion, epigenetic changes, loss of protein homeostasis, dysregulation of nutrient sensing, mitochondrial dysfunction, cellular senescence, and changes in intercellular communication (López-Otín et al., 2013). Among these, cellular senescence is one of the most important signs. Cellular senescence is a state characterized by long-term, irreversible cell cycle arrest, secretory features (senescence-associated secretory phenotype, or SASP), oxidative stress, macromolecular damage, and mitochondrial and lysosomal dysfunction (Gorgoulis et al., 2019). As aging progresses, neurons undergo iron deposition, lipid peroxidation, and autophagy disorders, which increase their sensitivity to ferroptosis (Coradduzza et al., 2023; **Figure 4**).

### Aging and iron overload

Iron is an important element in the physiological processes of the central nervous system and participates in many reactions, including oxidative metabolism, myelination, and neurotransmitter biosynthesis. Iron deposition in the brain has been shown to be associated with the onset of neurodegenerative diseases such as AD, PD), HD, and ALS (Dusek et al., 2022). Aging is an important risk factor for imbalances in iron homeostasis. Iron deposition in the nervous system is positively correlated with age, reaching its maximum level in the basal ganglia, including the putamen, globus pallidus, and substantia nigra (Ashraf et al., 2018). Additionally, ferritin levels in the frontal cortex, caudate nucleus, putamen, substantia nigra, and globus pallidus have been observed to increase with age. Neuromelanin serves as the most prominent iron storage site in the substantia nigra and locus coeruleus, and its expression increases with age (Tian et al., 2022). The specific mechanisms underlying aging-related brain iron deposition are not completely clear, but confirmed factors include cerebrovascular dysregulation, inflammation, increased blood-brain barrier permeability, and redistribution of iron in the brain (Grubić Kezele and Ćurko-Cofek, 2020). Microglia help maintain iron homeostasis in the brain by absorbing and storing iron molecules in ferritin (MacKenzie et al., 2008). However, in senescent microglia, overexpression of heme oxygenase-1 (HO-1) decomposes the heme group into iron, resulting in increased iron accumulation in the brain with age (Fernández-Mendívil et al., 2021). Moreover, as the brain ages, iron is partially converted from a stable form to iron hydroxide, which can induce oxidative stress in neurons (Weinreb et al., 2013). In conclusion, iron concentration in the brain increases with age, and iron overload induces oxidative reactions that heighten the brain’s sensitivity to ferroptosis.

Furthermore, aging-related iron deposition is also present in senescent cells. Cellular ferritin levels increase significantly with age, and high cellular ferritin levels are considered a characteristic of cellular senescence (Liu et al., 2019). Ferritin accumulates in the cytosol and mitochondria, and is subsequently degraded by autophagy to release unstable iron, thereby increasing the concentration of iron in the LIP (Zeidan et al., 2021). In senescent cells, iron concentration can increase tenfold, and iron exists in various forms, including lipofuscin and ferritin. Like ferritin, lipofuscin levels also increase with age, and elevated lipofuscin levels are considered a feature of cellular senescence (Xian-hui et al., 2015). Additionally, Xian-hui et al. (2015) confirmed that FPN1 expression decreases while DMT1 expression increases with age, both of which contribute to elevated ferrous iron concentration in senescent cells. Moreover, excessive iron deposition in senescent cells leads to the overproduction of ROS, a process known as ferrosenescence (Sfera et al., 2018). Interestingly, findings from two studies have confirmed that senescent cells often exhibit severe ferritinophagy defects, which can partially enhance their resistance to ferroptosis (Masaldan et al., 2018; Wei et al., 2023). However, we believe that while the ferritinophagy deficiency resulting from downregulated autophagy after aging may inhibit ferroptosis in senescent cells to some extent, this effect is insufficient to offset iron deposition that occurs with cellular senescence. Meanwhile, most studies support the notion that the primary manifestation of senescent cells is age-related iron overload (Xian-hui et al., 2015; Liu et al., 2019; Fernández-Mendívil et al., 2021).

It is worth noting that, after reviewing the relevant literature, we suggest that iron deposition in senescent cells may also trigger a series of chain reactions. Iron deposition can initiate oxidative stress and drive autophagy, thereby activating ferritinophagy. At the same time, iron accumulation in mitochondria leads to increased mitochondrial damage and oxidative stress, which in turn drives mitophagy. These chain reactions may form a feedback pathway that induces ferroptosis.

### Aging, lipid peroxidation, and the antioxidant system

Apart from brain iron deposition, lipid peroxidation has also been shown to be related to aging. Zeng et al. (2024) confirmed that lipid uptake is enhanced, lipid synthesis is accelerated, and lipid aggregation occurs in senescent cells. Compared with young men, middle-aged men have considerably greater levels of monounsaturated fatty acids and PUFA. Additionally, monounsaturated fatty acids and PUFA levels in the gray matter of the orbitofrontal cortex increase with age (Kao et al., 2020). One characteristic of cellular senescence is mitochondrial dysfunction, which leads to the accumulation of ROS and increases the sensitivity of cells to lipid peroxidation (Martini and Passos, 2023). Calcium (Ca^2+^) levels change with age, causing Ca^2+^ to target ROS generation sites, including mitochondrial respiration and nicotinamide adenine dinucleotide phosphate oxidase (NOX) enzymes, to manipulate ROS levels, ultimately inducing lipid peroxidation (Madreiter-Sokolowski et al., 2020). The levels of monoamine oxidase (MAO), especially MAO-B, increase with age, and MAO catalyzes the oxidative deamination of neurotransmitters. This reaction produces hydrogen peroxide, aldehydes, and other coproducts, all of which can induce lipid peroxidation (Santin et al., 2021). In senescent cells, the expression of Nrf2 decreases, thereby weakening the defense against lipid peroxidation (Zhang et al., 2024b). Nicotinamide adenine dinucleotide phosphate (NADPH), a reducing agent, is required for cellular defense against oxidative stress and is primarily synthesized when NAD^+^ is converted to NADP^+^ (Katsyuba et al., 2020). Moreover, NADPH can enhance the defense against ferroptosis through the NADPH-N-myristoyltransferase 2 (NMT2)-FSP1 axis (Liu et al., 2024b). NAD levels decrease with age (Imai and Guarente, 2014), as do NADPH levels, leading to a reduction in the antioxidant effect of NADPH with age. In summary, aging can promote the occurrence of ferroptosis through the activation of lipid peroxidation.

After correlating the research on post-aging peroxidation reactions with the aforementioned studies (Gao et al., 2016; Bi et al., 2024), further discoveries were made. Comprehensive research results indicate that peroxidation reactions can increase intestinal iron absorption and macrophage iron release by inhibiting hepcidin, releasing iron from oxidized damaged ferritin, and causing mitochondrial metabolic disorders that affect mitochondrial utilization and iron chelation. Additionally, the cell damage caused by the peroxidation reaction activates autophagy, which may further induce ferritinophagy and mitophagy, triggering ferroptosis and continuing to repeat until cell death occurs.

### Aging and autophagy

At appropriate levels, autophagy can eliminate harmful cellular components and help maintain cellular homeostasis. However, the overall level of autophagy decreases with age (Kitada and Koya, 2021). The levels of related proteins that play a role in the regulatory mechanism of autophagy fluctuate with age, and the translation and modification of autophagy proteins become dysregulated, ultimately leading to a decline in autophagy levels as aging progresses (Lim et al., 2024). The mechanism driving the decline in autophagy levels in aging cells is not entirely clear, but the increase in the number of autophagosomes alongside a decrease in flux suggests that the decline may occur in the later stages of autophagy. Numerous factors contribute to the downregulation of autophagy with age, including but not limited to changes in signaling pathways, as well as mitochondrial and lysosomal dysfunction. The silent information regulator sirtuin 1 (SIRT1) participates in autophagy-related signaling pathways and promotes autophagy, and a previous study has confirmed that SIRT1 levels significantly decrease with age (Mundo Rivera et al., 2024). Expression levels of autophagy-related proteins such as Atg5, Atg7, and Beclin 1 are downregulated in the aging brain (Tabibzadeh, 2023). Consequently, the protective effect provided by autophagy gradually diminishes with age. As organisms age, mitochondria become inefficient and dysfunctional, with these effects being particularly pronounced in the nervous system (Liang et al., 2021). Mitochondrial dysfunction activates mitophagy, and excessive clearance of mitochondria can lead to mitochondrial deficiency and increased levels of oxidative substances such as ROS (Li et al., 2022a). In addition to mitochondria, lysosomes are another important organelle involved in the autophagic process. Autophagy is a lysosome-based degradation process, and lysosomes become dysfunctional with age. Animal model studies mentioned in the review by Kaushik et al. (2021) have confirmed that the morphology and function of aging lysosomes change, including lysosomal compartment expansion, an increase in the levels of various lysosomal proteases, decreased activity of these proteases, and the accumulation of undegraded substances in lysosomes in the form of lipofuscin. However, the mechanisms behind aging-related lysosomal dysfunction proposed in the review by Guerrero-Navarro et al. (2022) include dysfunction of V-ATPase, lysosomal alkalinization, abnormal storage of lysosomal amino acids, ion disorders, and lipofuscin aggregation. It is well known that moderate autophagy levels positively influence the inhibition of ferroptosis (Chen et al., 2021c). Therefore, the decline in autophagic flux and the increase in aging-related oxidative reactions in senescent cells may trigger ferroptosis.

In addition to the direct research evidence mentioned above, we have drawn the following insights from an in-depth study of the relevant literature. On one hand, weakened autophagy after aging leads to excessive accumulation of lipid droplets and an increase in ROS in mitochondria, which may heighten sensitivity to ferroptosis. On the other hand, the decline in autophagy theoretically reduces the likelihood of ferritinophagy and mitophagy, potentially delaying the onset of ferroptosis to some extent. As mentioned earlier, the effect of autophagy on ferroptosis is bidirectional. However, most current studies support the idea that the aging process may increase cellular sensitivity to ferroptosis (Coradduzza et al., 2023b; Du et al., 2024). We believe that the degree of decline in ferritinophagy and mitophagy is insufficient to reverse the occurrence of ferroptosis. An overall decline in autophagy will weaken the ability to clear ferroptosis-activating substances, thereby resulting in a net positive regulation of ferroptosis.

### Ferroptosis in aging

There is a bidirectional interaction between aging and ferroptosis. Not only can aging induce ferroptosis, but ferroptosis has also been shown to play an important role in the aging process, with inhibiting ferroptosis emerging as a potential anti-aging strategy (Mazhar et al., 2021). Sun et al (2024a) confirmed that pro-ferroptotic signaling promotes vascular smooth muscle cell senescence, which in turn accelerates senescence in the cardiovascular system. In the research conducted by Wu et al. (2024), an imbalance in iron homeostasis and ferroptosis in macrophages were found to play significant roles in kidney senescence. Similarly, Wang et al. (2025b) demonstrated that the activation of ferroptosis leads to renal aging. Ferroptosis and its related genes are involved in all physiological processes of the nervous system, from development to functional maintenance and aging (Zhou et al., 2022). There is evidence that ferroptosis is closely related to brain aging (Yan et al., 2024).

Based on the evidence presented, ferroptosis will undoubtedly accelerate the aging process. Recent studies have confirmed the important role of inhibiting ferroptosis in delaying aging and prolonging the lifespan of many species (Fu et al., 2025). Melatonin, a ferroptosis inhibitor, has shown efficacy in delaying brain aging and nerve degeneration (Yehia and Abulseoud, 2024). Interestingly, nanoplasticsaccelerate placental senescence by inducing ferroptosis, whereas inhibiting ferroptosis can reverse nanoplastics-induced placental senescence (Chen et al., 2025b). Therefore, inhibiting ferroptosis is a promising anti-aging strategy.

## Ferroptosis and Aging-Related Neurodegenerative Diseases

With the extension of human life expectancy, the aging population is growing rapidly. By 2050, the number of individuals older than 60 years is expected to reach 2.1 billion (Amaya-Montoya et al., 2020). As the population ages, there is an increased incidence of age-related diseases, particularly neurodegenerative diseases such as AD, PD, HD, and ALS (Luo et al., 2020). It is well known that aging is a significant risk factor for these neurodegenerative diseases. Research has confirmed that iron accumulation, oxidative stress, and neural cell dysfunction and damage are key pathogenic mechanisms underlying age-related neurodegenerative diseases (Zhou et al., 2020b). Therefore, there are complex associations among aging, ferroptosis, and neurodegenerative diseases, and ferroptosis is likely involved in the pathogenesis of these conditions.

To investigate these hypotheses, we searched the GeneCards gene database. By entering the terms “aging-related neurodegenerative diseases,” “AD,” “PD,” “HD,” “ALS,” and “ferroptosis,” we obtained all related genes, including protein-coding genes, RNA genes, functional elements, pseudogenes, genetic loci, and gene clusters. We used a Boolean model to find matching documents, followed by a practical scoring function to calculate relevance, which was subsequently sorted to form the respective gene banks. The gene banks for the disease group were compared with those for the ferroptosis group to identify overlapping genes. All disease gene banks were combined for comparison to find genes that overlap across all diseases and ferroptosis.

**Figure 5:**
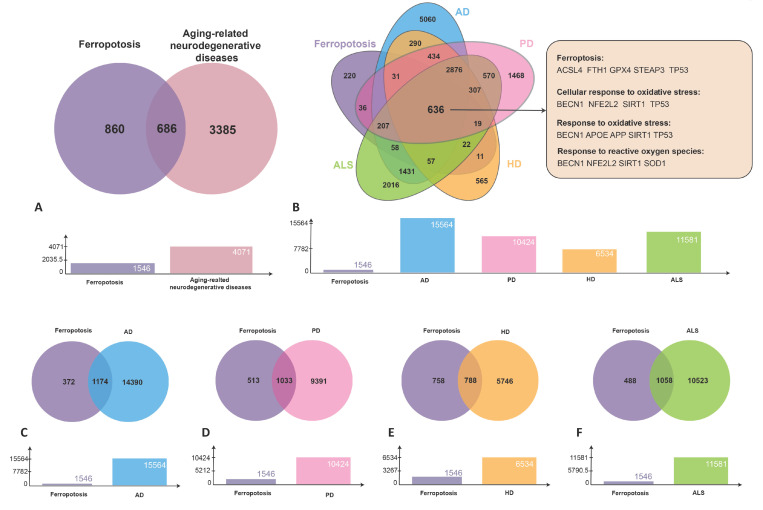
Genes related to aging-related neurodegenerative diseases and ferroptosis. (A) The GeneCards database was screened to select genes related to aging-related neurodegenerative diseases, including AD, PD, HD, ALS, and ferroptosis. (B) A Boolean model and a practical scoring function were used to calculate relevance and sort the results into respective gene banks. The high-ranking and node genes involved in this review are illustrated in panel B. The gene banks for the disease groups were individually compared with those of the ferroptosis group to identify overlapping genes. A combined comparison of all disease gene banks with the ferroptosis gene bank was conducted to identify common overlapping genes. (C–F) The coincidence rates of genes related to ferroptosis and aging-related neurodegenerative diseases were as follows: 686 genes (16.85%) for aging-related neurodegenerative diseases, 1174 genes (7.54%) for AD, 1033 genes (9.91%) for PD, 788 genes (12.06%) for HD, and 1058 genes (9.14%) for ALS. In total, 636 genes overlapped among all diseases and ferroptosis. Further functional network enrichment analysis of the 636 overlapping genes was performed using the STRING database, revealing that genes with close network connections and high node association strengths were enriched in the categories of cellular response to oxidative stress, response to oxidative stress, response to reactive oxygen species, and the ferroptosis pathway. ACSL4: Acyl-CoA synthetase long-chain family member 4; AD: Alzheimer’s disease; ALS: amyotrophic lateral sclerosis; ApoE: apolipoprotein E; APP: amyloid precursor protein; BECN1: beclin 1; FTH1: ferritin heavy chain 1; GPX4: glutathione peroxidase 4; HD: Huntington’s disease; NFE2L2: nuclear factor erythroid 2-related factor 2; PD: Parkinson’s disease; SIRT1: silent information regulator sirtuin 1; SOD1: superoxide dismutase 1; STEAP3: six-transmembrane epithelial antigen of prostate 3; TP53: tumor protein 53.

Through this analysis, we found the following numbers of overlapping genes (overlapping rates) among aging-related neurodegenerative diseases, AD, PD, HD, ALS, and ferroptosis: 686 (16.85%), 1174 (7.54%), 1033 (9.91%), 788 (12.06%), and 1058 (9.13%), respectively. In total, there are 636 genes that overlap between all the diseases and ferroptosis. The specific results are illustrated in the Venn diagram (**Figure 5**). The 636 overlapping genes were imported into the SRRING database for functional network enrichment analysis. We found that, in the biological process category of the Gene Ontology database, genes with close network connections and high node association strength are enriched in “cellular response to oxidative stress,” “response to oxidative stress,” and “response to reactive oxygen species.” The most enriched pathway identified in the KEGG database was the ferroptosis pathway. The genes that ranked highest in each section and are mentioned in this article are listed in **Figure 5B**.

### Ferroptosis and Alzheimer’s disease

**Figure 6:**
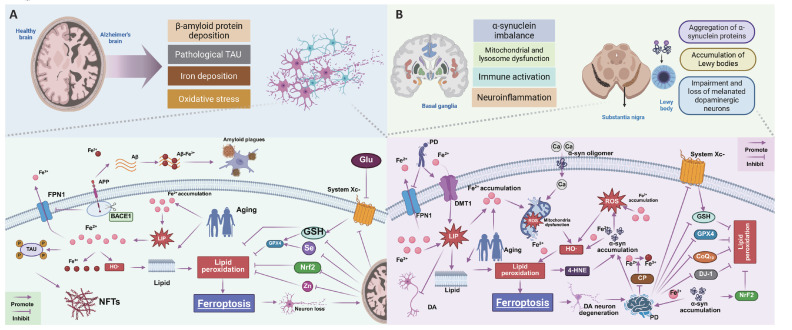
AD, PD, and ferroptosis. (A) AD and ferroptosis. After AD develops, neurons undergo iron overload and the cellular lipid antioxidant system is inhibited. Additionally, aging contributes to the accumulation of iron and lipids. These factors collectively promote the occurrence of ferroptosis, leading to neuronal loss and facilitating the onset and progression of AD. (B) PD and ferroptosis. During the development of PD, α-syn is synthesized. The oligomerization of α-syn triggers calcium influx, resulting in mitochondrial dysfunction and accelerating lipid peroxidation. Following the onset of PD, DA neurons experience decreased iron output and increased input, dysfunction of the cellular lipid antioxidant system, and age-related accumulation of iron and lipids. This results in ferroptosis of DA neurons, contributing to their degeneration and promoting the onset and progression of PD. Created with BioRender.com. 4-HNE: 4-Hydroxy-2-nonenal; AD: Alzheimer’s disease; APP: amyloid precursor protein; Aβ: amyloid beta; BACE1: beta-site amyloid precursor protein cleaving enzyme 1; CoQ10: coenzyme Q10; CP: ceruloplasmin; DA neuron: dopaminergic neuron; DMT1: divalent metal transporter 1; FPN1: ferroportin 1; Glu: glutamate; GPX4: glutathione peroxidase 4; GSH: glutathione; LIP: labile iron pool; NFTs: neurofibrillary tangles; NrF2: nuclear factor erythroid 2-related factor 2; PD: Parkinson’s disease; ROS: reactive oxygen species; Se:selenium; Zn: zinc; α-syn: alpha-synuclein.

AD is the leading cause of dementia and is considered one of the most expensive and fatal diseases of this century. In 2018, there were approximately 50 million patients with AD worldwide, and this number is expected to triple by 2050, with two-thirds of AD patients residing in low- or middle-income countries (Scheltens et al., 2021). Currently, the widely accepted diagnostic criteria for AD are based on the ATN framework: Aβ aggregation and the related pathological state (A); phosphorylated tau protein and neurofibrillary tangles (NFTs) along with their associated pathological state (T); and neurodegeneration and neuronal injury (N). The presence of both Aβ and phosphorylated tau can be used to diagnose AD (Jack et al., 2018). Although the formation of Aβ plaques and NFTs may not be the underlying mechanisms of AD pathogenesis, abnormal protein deposition distinguishes AD from other neurodegenerative diseases that can also lead to dementia. In addition to the ATN framework, AD is associated with cognitive impairment (**Figure 5C**). Neurodegeneration and cognitive impairment are not specific changes to AD; therefore, these factors are only used to evaluate the severity of the disease. Studies suggest that iron overload and oxidative stress may be contributing factors in AD, and the application of iron chelators as well as antioxidants can effectively mitigate cognitive impairment in patients with AD (Plascencia-Villa and Perry, 2021; Wang et al., 2022c). Consequently, ferroptosis may represent one of the pathogenic mechanisms underlying AD (**Figure 6A**).

Previous studies have shown that iron homeostasis in the brain is severely disrupted after the onset of AD (Lei et al., 2021; Ma et al., 2022). Examination of brain tissue post-AD development has revealed numerous pathological changes consistent with ferroptosis (Stockwell et al., 2017). Biochemical changes indicative of ferroptosis in AD, including downregulation of GPX4, depletion of GSH, global redox disorders, significant lipid peroxidation, and mitochondrial dysfunction, have been observed *in vivo*, *in vitro*, and in clinical studies, as noted in the review by Plascencia-Villa and Perry (2021). Currais et al. (2025) evaluated the occurrence of ferroptosis in the AD brain by analyzing the transcriptomic characteristics of ferroptosis in AD cells, animal models, and human AD brain samples. Their research supports the involvement of ferroptosis in AD. Autopsies of AD patient brain tissue revealed increased levels of iron, TF, and ferritin, with significant iron accumulation observed in the frontal cortex and hippocampus—regions most affected by AD (Lei et al., 2021; Ma et al., 2022). Iron deposition occurs in the cerebral cortex and hippocampus following AD development, exacerbating the deposition of Aβ (Ayton et al., 2020; Xu et al., 2020). Iron overload can also lead to abnormal phosphorylation of tau in neurons (Wan et al., 2019). After AD develops, APP is hydrolyzed and cleaved into Aβ, and the deposition of Aβ results in dysfunction of FPN1, which further exacerbates neuronal iron overload (Belaidi et al., 2018; Tsatsanis et al., 2020). Findings from studies (Raha et al., 2013; Bao et al., 2021) also confirmed that FPN1 levels are significantly decreased in AD mouse models and patients, suggesting that this decrease plays a key role in neuronal loss and memory impairment after the onset of AD. Additionally, there was no significant change in the mRNA levels of FPN1 in patients with AD, which may indicate post-transcriptional dysregulation. A literature review suggested that this could be due to the specific upregulation of miR-124 in the hippocampus and temporal cortex following AD (Bao et al., 2021). In addition to FPN1, mitochondrial ferritin, TF, and hepcidin have also been shown to be dysregulated after AD develops, increasing susceptibility to ferroptosis (Jakaria et al., 2021). After the onset of AD, levels of HO-1 increase in astrocytes within brain tissue, accelerating the decomposition of heme into free Fe^2+^ (Schipper et al., 2019). Furthermore, our comprehensive study suggests that iron overload can also increase ROS production by damaging mitochondria, leading to peroxidation reactions, further consumption of antioxidant substances, and the induction of ferroptosis.

The abnormal activation of ferritinophagy will undoubtedly increase the probability of ferroptosis, and inhibiting ferritinophagy is expected to become one of the breakthrough points in ADtreatment. Research by Santana-Codina et al. (2021) has confirmed that ferritinophagy is highly dependent on nuclear receptor coactivator 4 (NCOA4), which finely regulates cellular iron homeostasis by modulating the autophagic degradation of ferritin. When cells are iron deficient, ferritin light chain vesicles increase cellular iron content through NCOA4-mediated ferritinophagy (Lee and Hyun, 2023). Therefore, NCOA4-mediated ferritinophagy has great potential as a therapeutic intervention for AD (Chen et al., 2025c). Mutations that reduce tau lactylation at the K677 site inhibit ferritinophagy and subsequently inhibit ferroptosis by regulating factors such as NCOA4 and FTH1, ultimately improving the cognitive function of patients with AD (An et al., 2024). As we know, variations in the apolipoprotein E (ApoE) gene are a risk factor for AD. A low concentration of ApoE in cerebrospinal fluid is a characteristic of AD and is often accompanied by cognitive impairment in patients. However, ApoE itself is a potent inhibitor of ferroptosis, activating the phosphatidylinositol 3-kinase (PI3K)/protein kinase B (PKB/AKT) pathway to inhibit ferritinophagy, thereby reducing iron-dependent lipid peroxidation (Belaidi et al., 2024).

In addition to iron overload, oxidative stress and lipid peroxidation have also been shown to be associated with the pathogenesis of AD. The sources of ROS in brain tissue are diverse, and the oxidative capacity is strong, but the antioxidant capacity is limited (Mantha et al., 2006). Neuronal membranes are rich in PUFAs, and considerably increased levels of lipid peroxidation markers have been detected in patients with AD, especially in the frontal, parietal, and temporal lobes (Ansari and Scheff, 2010). LOX catalyzes lipid peroxidation and is an important catalyst for ferroptosis (Lee et al., 2021). The activity of 12/15-LOX is upregulated in the AD brain and is associated with the oxidation of phospholipids (Plascencia-Villa and Perry, 2021). NADPH oxidase 4 impairs mitochondrial metabolism after AD develops by inhibiting mitochondrial respiration and ATP production, which in turn induces lipid peroxidation and leads to ferroptosis in astrocytes (Park et al., 2021). Based on autopsy results of patients with AD, *ApoE-ε4* gene carriers have high PUFA levels and are more susceptible to ferroptosis (Belaidi et al., 2024). Additionally, lipid peroxidation is considered to be upstream of Aβ, as it can cause Aβ aggregation after the onset of AD (Arimon et al., 2015). Previously, we reported that the deposition of Aβ leads to functional impairment of FPN, exacerbating iron overload in neurons. One of the products of lipid peroxidation, 4-HNE, can produce Aβ fibrils and NFTs in AD (Lee and Hyun, 2023). Tau aggregation after the development of AD induces iron overload and lipid peroxidation (Zhang et al., 2018), leading to ferroptosis and accelerating disease progression.

As antioxidants, GSH and GPX4 are significantly downregulated in the frontal cortex and hippocampus after the onset of AD and are positively correlated with disease severity (Ansari and Scheff, 2010; Mandal et al., 2015; Shukla et al., 2020). GPX4 levels are reduced in AD model mice, likely due to the downregulation of guanine-rich RNA sequence binding factor 1, which regulates GPX4 translation (Yoo et al., 2010). The decreased expression of glutamate (Glu) transporters in the cerebral cortex of patients with AD leads to the accumulation of Glu outside nerve cells, inhibiting system XC^−^ (Ma et al., 2022). This inhibition results in GPX4 downregulation and LOX activation, thereby inducing ferroptosis. As mentioned above, when system XC^−^ is inhibited, autophagy is activated by reducing the concentration of cysteine, which further promotes ferroptosis. Zinc (Zn) has antioxidant effects, and studies have shown a significant reduction in brain Zn levels in patients with AD (Ashraf et al., 2020; Chen et al., 2025a). In addition to Zn, selenium (Se) is also an antioxidant that can increase the expression of GPX4 (Alim et al., 2019). Findings from a cohort study confirmed that the plasma Se concentration in AD patients was lower than that in healthy controls (Cardoso et al., 2010). Iron overload occurs in nerve cells after the onset of AD, and the excess iron is undoubtedly one of the important factors affecting Se expression.

Transcription factor levels are similarly dysregulated in AD. It is well known that Nrf2 is a negative regulator of ferroptosis and serves as a protective factor against AD pathological changes. Reduced expression of Nrf2 has been observed in both aging AD animal models and the brains of AD patients (Bahn and Jo, 2019). In addition, recent studies have shown that ATFs are involved in the pathogenesis of AD, with differing expression levels of various ATFs in AD (Yin et al., 2019; Yang et al., 2023c; Goswami et al., 2024). Among these, ATF4 has been shown to be elevated in AD models and to induce cell death by mediating ER stress (Goswami et al., 2024). ATF3 is upregulated during nerve injury and is associated with neuronal oxidative stress, mitochondrial dysfunction, and ER stress (Yin et al., 2019). In conclusion, we speculate that ATFs can induce ferroptosis through oxidative stress during AD. As a protein encoded by TP53, p53 mediates ferroptosis through the p53/SAT1/arachidonate 15-lipoxygenase (ALOX15) signaling pathway, which is activated in AD, leading to cognitive impairment (Ren et al., 2024). As a gene encoding p53, TP53 has been indirectly confirmed by the above studies to be involved in mediating ferroptosis in AD.

As mentioned above, the regulation of ferroptosis by transcription factors depends on downstream factors and is bidirectional. Therefore, reversing ferroptosis in AD by regulating transcription factors holds potential for development. Artemisinin activates the Nrf2/SLC7A11/GPX4 pathway by promoting the dissociation of the KEAP1-Nrf2 complex and inhibiting Nrf2 ubiquitination, thereby reversing ferroptosis in AD (Deng et al., 2025). Ganoderic acid A can also inhibit ferroptosis by activating the Nrf2/SLC7A11/GPX4 pathway and reducing hippocampal neuronal damage in AD model mice (Lu et al., 2025). Additionally, downregulation of ATF4 has been shown to alleviate cognitive impairment following AD (Goswami et al., 2024). Cerebroprotein hydrolysate-I ameliorates cognitive impairment in AD mice by inhibiting ferroptosis via the p53/SAT1/ALOX15 signaling pathway (Ren et al., 2024). These studies not only confirm the therapeutic potential of regulating transcription factors but also further establish the existence and important role of ferroptosis in AD (Goswami et al., 2024; Ren et al., 2024; Deng et al., 2025).

### Ferroptosis and Parkinson’s disease

PD is the second most common neurodegenerative disease after AD (Gu et al., 2024; Huenchuguala and Segura-Aguilar, 2024), with approximately 6.06 million people worldwide suffering from PD as of 2016 (GBD 2016 Neurology Collaborators, 2019). Patients with PD primarily present with motor dysfunction, including resting tremor, rigidity, and bradykinesia, as well as non-motor symptoms such as sleep disturbances, autonomic dysfunction, cognitive impairment, and psychiatric symptoms (Dovonou et al., 2023). The characteristic pathological changes of PD include the degeneration and death of DA neurons in the substantia nigra and other regions of the brain, the misfolding and aggregation of α-syn, and the formation of Lewy bodies (Marogianni et al., 2020). Aging is the most significant risk factor for PD (Tolosa et al., 2021). The pathogenic mechanisms underlying PD are complex and not fully understood, involving α-syn imbalance, mitochondrial and lysosomal dysfunction, immune activation, and neuroinflammation (Morris et al., 2024).

Increased levels of iron and lipid oxidation products have been observed in the substantia nigra pars compacta (SNPC) of brains affected by PD (Ma et al., 2021), whereas the inhibition of ferroptosis has been demonstrated to ameliorate PD symptoms (Li et al., 2023a). Mutations in several ferroptosis-related genes are associated with PD, and the characteristics of ferroptosis are highly consistent with the pathological changes observed in PD patients, including increased iron concentrations, markers of lipid peroxidation, and defects in the antioxidant system (Wang et al., 2022f). These findings suggest that ferroptosis may be one of the pathogenic mechanisms of PD (**Figure 6B**).

Iron overload in patients with PD has been shown to be associated with increased iron input and decreased output. Findings from PD animal and cell models in the review by Ma et al. (2021) confirmed that DMT1 upregulation and FPN1 downregulation after PD development result in iron overload. APP can stabilize FPN1 on the cell surface to export iron; however, the expression of APP decreases after PD develops. The expression of transferrin receptor 2 (TfR2) is increased, and the Tf/TfR2 complex helps transport iron into the mitochondria of SNPC neurons, thereby increasing the likelihood that iron triggers oxidative stress (Duță et al., 2024). Ferritin and neuromelanin are commonly used to store iron, but they become saturated in PD, resulting in excess iron being released into the LIP (Hare and Double, 2016). Iron and α-syn coexist in the Lewy bodies of patients with PD, and α-syn can bind with overloaded iron, altering its structure and promoting aggregation, thereby aggravating PD symptoms (Dong-Chen et al., 2023). Additionally, in the early stages of PD, misfolded α-syn activates autophagy. Based on existing research, once autophagy is activated, it leads to ferritinophagy and mitophagy, further inducing ferroptosis. Furthermore, iron overload can increase the oxidation of dopamine, leading to a reduction in DA neurons in PD. The ferritase activity of ceruloplasmin (CP) can oxidize Fe^2+^ to Fe^3+^, but CP activity is reduced in approximately 80% of patients with PD (Dong-Chen et al., 2023). Moreover, glial cells, including astrocytes, microglia, and oligodendrocytes, play key roles in regulating iron homeostasis. Glial cells are rich in various iron-related proteins (DMT1, FPN1, CP) and are responsible for the import, utilization, storage, and export of iron. Abnormalities in glial cells following PD development may lead to iron metabolism disorders and the degeneration of DA neurons (Xu et al., 2017).

Ferritinophagy has been shown to play a role in PD by mediating ferroptosis, with the mechanism involving NCOA4, ferritin within autophagosomes, and lysosomal proteolysis activity (Yao et al., 2024b). Among these mechanisms, NCOA4 appears to dominate and is the most widely studied. Therefore, inhibiting NCOA4 to reverse the ferroptosis of DA neurons after PD has clinical prospects. Following PD, DA neurons are particularly susceptible to ferroptosis during the disease’s progression, which is attributed to the enhancement of ferroptophagy mediated by NCOA4. In contrast, JWA can alleviate the ferroptosis of DA neurons by occupying the ferritin binding site of NCOA4 (Zhao et al., 2024). Ganoderic acid A can also protect DA neurons from ferroptosis by inhibiting NCOA4-mediated ferritinophagy (Li et al., 2024). Additionally, clozapine-N-oxide (CNO) has been shown to protect DA neurons by regulating NCOA4-mediated ferroptophagy against ferroptosis (Sun et al., 2024b). Furthermore, Dev et al. (2025) confirmed that DA cells degrade ferritin via CMA to elevate mitochondrial iron levels in astroglial cells, thereby triggering dysregulation of iron homeostasis and mitochondrial dysfunction in PD.

Interestingly, ferritinophagy may be inhibited by α-syn aggregation after PD develops (Boag et al., 2022). It is well known that brain iron deposition is an important manifestation of PD. Therefore, after comprehensively considering the conclusions of relevant studies, we believe that α-syn generates ER stress and affects the fusion of ferritin and lysosomes in autophagosomes by preventing vesicle transport (Mazzulli et al., 2016; Nascimento et al., 2020). However, excessive intracellular iron is stored in ferritin, and autophagosomes containing ferritin may suffer internal redox damage due to ferritin denaturation if they are overloaded for a prolonged period. Ultimately, autophagosomes will rupture and release their contents, further leading to redox-dependent ferroptosis. Additionally, the excessive aggregation of α-syn in the late stages of PD, which inhibits autophagy, may also contribute to this process. NOX1 catalyzes the reaction between NADPH and oxygen molecules to produce ROS in neurons. Furthermore, NOX1 has been shown to activate ferroptosis by promoting ferritinophagy, which, in turn, leads to the loss of dopaminergic neurons and the progression of PD (Wang et al., 2024c). FTH1 attenuates susceptibility to ferroptosis by inhibiting ferritinophagy and mitochondrial damage (Tian et al., 2020).

Oxidative stress plays an important role in PD by causing oxidative damage to lipids, proteins, DNA, and RNA. This oxidative stress leads to the degeneration of DA neurons, which can be observed before neuronal death occurs (Trist et al., 2019). The neurons in the brain tissue of patients with PD are rich in PUFAs, which have strong oxidative capacity but limited antioxidant capacity. Dexter et al. (1986) reported a decrease in PUFA levels and significant increases in MDA and lipid peroxide levels in the substantia nigra of patients with PD, as observed through autopsy in the 1980s. Subsequent studies confirmed the significant increase in lipid peroxidation markers following the induction of PD. At the same time, ACSL4 and ALOX15(B), key regulators of lipid peroxidation, have been shown to be involved in RSL3-induced ferroptosis of DA neurons (Su et al., 2024). It is unequivocally established that lipid peroxidation is closely related to PD. In the initial stages of PD, 4-HNE, a product of lipid peroxidation, promotes α-syn aggregation (Lee and Hyun, 2023). The aggregation of α-syn can also produce ROS, which induce lipid peroxidation, leading to increased calcium influx and ferroptosis in cells (Angelova et al., 2020). Lipid peroxidation products, such as 4-HNE, can covalently modify α-syn, producing harmful substances that further damage cells (Shamoto-Nagai et al., 2018). Additionally, as mentioned previously, α-syn aggregation may activate autophagy, inducing ferritinophagy and mitophagy. Therefore, lipid peroxidation and iron-mediated ferroptosis are primary causes of neural cell death in patients with PD and may participate in the pathogenic mechanisms of PD.

In the SNPC of patients with PD, the levels of antioxidants such as GSH and GPX4 are decreased (Bjørklund et al., 2021; Ma et al., 2021). Hypermethylation of the Cg06690548 locus on chromosome 4 in PD leads to the downregulation of SLC7A11 gene expression and, ultimately, a reduction in system Xc^–^ expression (Vallerga et al., 2020). One study revealed that patients with PD have a greater probability of coenzyme Q10 deficiency (Mischley et al., 2012). The downregulation of the above antioxidant systems after the development of PD may increase the risk of ferroptosis. Mutations in the DJ-1 protein can cause autosomal-recessive PD (Skou et al., 2024). Additionally, DJ-1 has antioxidant effects and can inhibit ferroptosis through the transsulfuration pathway, compensating for intracellular cysteine depletion when cystine import is inhibited (Cao et al., 2020). After comprehensively considering the conclusions of previous research, we found that the downregulation of GSH and GPX4 after PD accelerates the consumption of cysteine, thereby increasing dependence on the antioxidant effect of DJ-1. However, elevated levels of N-homocysteinylation after PD development lead to the covalent modification of the lysine 182 (K182) residue of DJ-1 in an aging-dependent manner, inhibiting its antioxidant activity (Guo et al., 2024). In addition to the above direct evidence, GCH1 is a gene related to both PD and ferroptosis, and its mutation or abnormal expression may affect PD by mediating ferroptosis (Chu et al., 2023a).

Ferroptosis in PD is also regulated by transcription factors and noncoding RNAs. Among these, Nrf2 is an important transcription factor that regulates cellular defense mechanisms, including neuroinflammation, oxidative stress, and mitochondrial dysfunction, playing a crucial role in protecting DA neurons from ferroptosis and preventing PD (Kaur and Aran, 2025). The overexpression of α-syn after the onset of PD induces DA neuron death by inhibiting Nrf2 (Pandey et al., 2021), whereas the activation of Nrf2 to inhibit ferroptosis can protect DA neurons from death (Lin et al., 2022). Additionally, mitochondrial damage is a primary cause of DA neuron degeneration in PD, and mitochondrial dysfunction-related degeneration is strictly regulated by p53 in the pathogenesis of PD. Therefore, the activation of p53 is closely associated with the occurrence and progression of PD (Wang et al., 2024e). P53 has also been confirmed to be involved in ferroptosis-related signaling pathways in PD (Yao et al., 2024a). Thus, we can infer that TP53 regulates ferroptosis-related signaling pathways in PD by encoding p53. In addition to transcription factors, noncoding RNAs, especially microRNAs, have been shown to play a role in regulating ferroptosis in PD. In PD rat and cell models, the overexpression of miR-335 can exacerbate ferroptosis and pathological changes associated with PD (Li et al., 2021). Furthermore, Li and Guo (2024) reported through bioinformatics analysis of ferroptosis-related genes that miR-26b-5p is associated with ferroptosis in PD.

As in AD, regulating transcription factors and their pathways to inhibit ferroptosis is one of the potential therapies for PD. Lv et al. (2024) reported that melatonin receptor 1 (MT1) prevents α-syn-induced ferroptosis in PD by activating the Sirt1/Nrf2/HO-1/Gpx4 pathway. Granulatiazole A inhibits ferroptosis by activating the Nrf2/HO-1 pathway and improves α-syn accumulation in a PD model (Kong et al., 2024). Acteoside attenuates neurotoxicity in PD by inhibiting ferroptosis through the activation of the Nrf2/SLC7A11/GPX4 pathway (Wang et al., 2025a). At the same time, acteoside can also reduce lipid peroxidation by enhancing Nrf2-mediated mitophagy, thereby inhibiting ferroptosis and exerting neuroprotective effects in PD (Han et al., 2024). Li et al. (2025) demonstrated that inhibiting p53 expression can effectively alleviate ferroptosis in PD.

### Ferroptosis and Huntington’s disease

**Figure 7:**
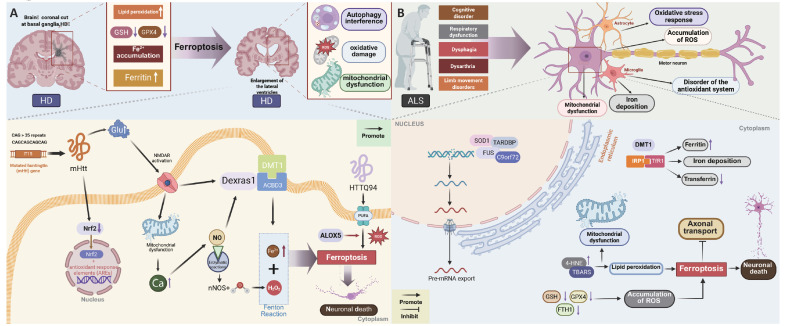
HD, ALS, and ferroptosis. (A) HD and ferroptosis. The abnormal expansion of CAG trinucleotide repeats gives rise to mHTT, which triggers ferroptosis via a series of intricate reactions. ALOX5 mediates the ACSL4-independent ferroptosis induced by HTTQ94. Additionally, iron deposition, elevated lipid peroxidation, reduced antioxidant capacity, and interference with autophagy can also lead to ferroptosis. Consequently, ferroptosis constitutes an essential neuronal cell death mode in HD. (B) ALS and ferroptosis. Iron overload within neurons and glial cells, mitochondrial dysfunction, the oxidative stress response, as well as the disorders of the antioxidant system, may mediate ferroptosis. Ferroptosis can further result in the death of neurocytes and dysfunction of axonal transport in ALS. Created with BioRender.com. ACBD3: Acyl-CoA-binding domain-containing 3; ALOX5: arachidonate 5-lipoxygenase; ALS: amyotrophic lateral sclerosis; C9orf72: chromosome 9 open reading frame 72; Dexras1: dexamethasone-induced Ras-related protein 1; DMT1: divalent metal transporter 1; FTH1: ferritin heavy chain 1; FUS: fused in sarcoma; Glu: glutamate;GPX4: glutathione peroxidase 4; GSH: glutathione; HD: Huntington’s disease; H2O2: hydrogen peroxide; IRP1: iron-responsive element–binding proteins 1; mHTT: mutant Huntingtin; Nrf2: nuclear factor erythroid 2-related factor 2; NO: nitric oxide; PUFA: deuterated polyunsaturated fatty acids; ROS: reactive oxygen species; SOD1: superoxide dismutase 1; TARDBP: TAR DNA-binding protein; TBARS: thiobarbituric acid reactive substances; TfR1: transferrin receptor 1; 4-HNE: 4-hydroxynonenal.

HD is an autosomal-dominant neurodegenerative disorder (Yu et al., 2024). Abnormal amplification of more than 35 cytosine-adenine-guanine (*CAG*) trinucleotide repeats in the coding region of the huntingtin (*HTT*) gene, located on chromosome 4, can lead to HD; the abnormally amplified HTT is referred to as mutant HTT (mHTT) (Gusella et al., 2021). The global prevalence of HD is approximately 2.7 per 100,000 (Pan and Feigin, 2021). Although the incidence of HD is much lower than that of AD and PD, HD often results in catastrophic and progressive motor deficits, psychiatric symptoms, and cognitive impairment, with a median survival of approximately 18 years after onset (Kim et al., 2021). In patients with HD, many manifestations similar to those of ferroptosis have also been observed, including increased levels of lipid peroxidation markers, decreased levels of GSH and GPX4 activity, and iron deposition in the putamen, globus pallidus, and occipital cortex; meanwhile, ferritin levels in the striatum are significantly increased (Mi et al., 2019). In a mouse model of HD, inhibiting lipid peroxidation and applying the iron chelator deferoxamine improved neurological function, while iron supplementation exacerbated neurological dysfunction (Mi et al., 2019; **Figure 7A**).

The production of mHTT is the main pathogenic mechanism of HD. In addition to altering the physiological effects of normal HTT, mHTT also exerts toxic effects. Based on comprehensive research, we summarize that mHTT may cause ROS accumulation by damaging mitochondria, inducing ferroptosis through interference with iron homeostasis regulatory proteins (such as IRP and FPN), inhibiting system Xc^–^ to deplete GSH, and activating autophagy; these factors work together to induce ferroptosis (Fields et al., 2021; Alkanli et al., 2023; Muller and Leavitt, 2014). Song et al. (2023b) reported that under the oxidative stress conditions associated with HD, arachidonate 5-lipoxygenase (ALOX5) can mediate the N-terminal fragment of mHTT with the expanded polyglutamine (HTTQ94) to activate ACSL4-independent neuronal ferroptosis, independent of GPX4 function.

Magnetic resonance imaging (MRI) examinations have revealed that iron concentration in patients with HD increases in the early stages of the disease, prior to the onset of clinical symptoms, and that iron deposition gradually increases with disease progression (Rosas et al., 2012). Subsequent studies confirmed that changes in iron levels play an important role in the pathological process of HD, occurring earlier than changes in brain structure. An animal model study showed that iron accumulation and progressive volume reduction occur in the striatum in the early stages of HD, while the iron concentration in the basal ganglia in the late stages is significantly greater. Additionally, autopsies of patients with advanced HD revealed increased iron concentrations in the caudate nucleus, putamen, and globus pallidus (van Bergen et al., 2016). Mitochondria are also key organelles involved in the iron dysregulation that occurs in HD, with unstable iron accumulating in HD mitochondria as the disease progresses (Agrawal et al., 2018). The increased number of ferritin-containing microglia in the brains of patients with HD suggests that iron overload in HD is related to microglia and occurs early, which may be a contributing factor in the progression of the disease (Simmons et al., 2007).

High levels of lipid peroxidation markers are another major feature of HD (Hatami et al., 2018). Autopsy studies revealed that GSH levels in the brain plasma of patients with HD were decreased, while lipid peroxidation marker levels were increased (Klepac et al., 2007). Additionally, increased concentrations of 4-HNE have been observed in the striatum of both patients with HD and HD model mice, and the administration of lipid peroxidation inhibitors can effectively prevent neuronal death (Lee et al., 2011). Findings from studies using rat HD models confirmed that Nrf2 expression decreased after the onset of HD, which in turn led to a reduction in antioxidant capacity (Sayed et al., 2020; Chan et al., 2023). Unfortunately, we did not find evidence that TP53 (p53) and ATF proteins regulate ferroptosis in HD.

### Ferroptosis and amyotrophic lateral sclerosis

ALS is a relatively rare but fatal neurodegenerative disease (Huber and Wang, 2024; King, 2024). The standardized incidence of ALS worldwide in 2016 was approximately 1.68 per 100,000 individuals (Marin et al., 2017). The incidence of ALS increases with age, peaking between 60 and 79 years, and then decreases thereafter (Marin et al., 2018). ALS is defined as a progressive, painless, and random muscle weakness disease with significant heterogeneity in terms of affected sites and clinical manifestations (Goutman et al., 2022). It primarily manifests as dysfunction of upper motor neurons (UMNs) and lower motor neurons (LMNs), including, but not limited to, limb dyskinesia, dysphagia, dysarthria, and progressive weakness of respiratory muscles (Feldman et al., 2022). In addition to motor dysfunction, ALS can also present as cognitive and executive dysfunction (Ilieva et al., 2023). Genetic factors are considered the most important cause of ALS. To date, more than 30 ALS-related genes, including superoxide dismutase 1 (SOD1), TAR DNA-binding protein (TARDBP), fused in sarcoma (FUS), and chromosome 9 open reading frame 72 (C9orf72), have been identified (Akçimen et al., 2023). Although the pathogenic mechanisms of ALS remain unclear, proposed mechanisms include impaired RNA metabolism, dysregulation of protein homeostasis and autophagy, cytoskeletal defects, blockade of synaptic transport, and mitochondrial dysfunction (Feldman et al., 2022).

The levels of ferroptosis-related markers, such as ferritin and 4-HNE, fluctuate significantly after the onset of ALS and are positively correlated with disease progression (Devos et al., 2019). Additionally, iron deposition, ROS aggregation, oxidative stress responses, and disorders of the antioxidant system have been shown to play a role in ALS (Wang et al., 2024d), and ferroptosis has been observed in ALS cell models (Wang et al., 2023b). Most importantly, characteristic mitochondrial changes associated with ferroptosis have been detected via transmission electron microscopy of spinal cord neurons in ALS model mice (Yang et al., 2023a; **Figure 7B**).

Findings from studies in an ALS mouse model and MRI of patients with ALS confirmed that iron accumulation primarily occurs in neurons and glial cells (Jeong et al., 2009; Bhattarai et al., 2022). In glial cells, the increase in TfR1 expression mediated by enhanced IRE-binding protein 1 (IRP1) binding activity leads to iron accumulation, which is mainly manifested by an increase in ferritin levels in microglia. An increase in DMT1 expression, to a much greater extent than FPN1 expression, exacerbates iron accumulation in neurons. A follow-up study revealed that serum ferritin levels in patients with ALS increased as TF levels decreased, and elevated serum ferritin levels were associated with faster disease progression (Wang et al., 2020a). A quantitative susceptibility MRI evaluation indicated that iron load increased in multiple brain tissue regions, most notably the motor cortex, substantia nigra, globus pallidus, red nucleus, and putamen, following the onset of ALS (Acosta-Cabronero et al., 2018). After treatment with deferiprone (DFP) at a dose of 30 mg/kg per day, a significant decrease in iron concentration was observed in the cervical spinal cord, medulla oblongata, and motor cortex of patients with early ALS, while iron levels in the cerebrospinal fluid and levels of oxidative stress markers significantly decreased (Moreau et al., 2018). Iron accumulation after the development of ALS can lead to lipid peroxidation, which, in turn, may result in ferroptosis. Additionally, impairment of the AKT signaling pathway following ALS development may contribute to iron metabolism disorders. A study using a mouse model by Halon-Golabek et al. (2024) revealed that the progression of ALS leads to a reduction in the p-AKT/AKT ratio, which in turn increases ferritin levels in skeletal muscle, while the level of iron export protein is significantly reduced.

Lipid peroxidation and mitochondrial dysfunction are involved in the pathogenesis of ALS, providing evidence for ferroptosis as a pathogenesis of ALS. An early study confirmed that lipid peroxidation occurred in the spinal cord of patients with ALS (Pedersen et al., 1998), and subsequent study findings also confirmed the occurrence of lipid peroxidation in blood samples from patients with ALS (Blasco et al., 2017). From the early stage to 3 months after the onset of ALS, the levels of lipid peroxidation products such as thiobarbituric acid reactive substances and 4-HNE increase, the levels of antioxidants such as GSH and GPX4 decrease, and astrocytes treated with plasma from patients with ALS exhibit decreased cell viability and mitochondrial membrane potential (Grossini et al., 2023). The application of antioxidants in ALS animal models and patients can improve neurological function and outcomes by reducing ROS production, regulating mitochondrialfunction and inhibiting lipid peroxidation (Carrera-Juliá et al., 2020).

Wang et al. (2022d) reported decreased GPX4 expression in the spinal cords of patients with ALS. Meanwhile, FTH1, TfR1, and GPX4 expression were downregulated in the spinal cords and brains of ALS model mice; Nrf2 expression was also downregulated in the spinal cords of ALS model mice. Additionally, GPX4 overexpression reversed the outcomes observed in the ALS model mice. Yang et al. (2023a) also confirmed that Nrf2 expression decreased after ALS development, and the restoration of Nrf2 expression could play a neuroprotective role by inhibiting ferroptosis. Similar to findings in HD, we did not find evidence that TP53 (p53) and ATFs regulate ferroptosis in ALS. GSH levels are reduced in ALS model mice, which in turn leads to the generation of ROS and an increase in the oxidative stress response, ultimately resulting in neuronal death (Chi et al., 2007). Speedy/RINGO cell cycle regulator family member A (SPY1) inhibits iron-induced lipid peroxidation in ALS by restoring GCH1/BH4 axis activity and downregulating TfR1 expression (Wang et al., 2023b). A decrease in SLC7A11 expression after ALS development leads to an imbalance in redox reactions and increases lipid peroxidation marker levels, which ultimately results in ferroptosis (Ismail et al., 2024). This research conclusion also confirms findings from previous studies (Chi et al., 2007; Wang et al., 2022d).

### Correlations and contradictions between researches

Based on the research conclusions presented, we found that ferroptosis can lead to neuronal cell death in various neurodegenerative diseases, resulting in neurological dysfunction and disturbances of consciousness. The mechanisms of ferroptosis in these diseases are complex and vary, but they are generally associated with age-related iron accumulation, enhanced peroxidation, mitochondrial dysfunction, autophagy levels, and disorders of transcription factors. Through the analysis and comparison of studies, we believe that there are many commonalities in the mechanisms and pathways of AD, PD, HD, and ALS. Proteins and factors such as GPX4, GSH, SLC7A11, Nrf2, and FPN1 have been frequently mentioned, while others, including DMT1, ALOX15, ATFs, and p53, have also been noted several times. At the same time, the mechanisms related to ferroptosis differ among these diseases and are often associated with characteristic proteins or genes specific to each condition. For example, Aβ and ApoE in AD, as well as APP and α-syn in PD, have been shown to regulate ferroptosis through their respective pathways. However, for HD and ALS, due to the limited number of relevant studies, characteristic mechanistic pathways remain elusive.

Although ferroptosis has been confirmed to lead to neurological dysfunction and disturbances of consciousness in patients with neurodegenerative diseases, it is important to summarize that, in these conditions, ferroptosis is induced and exacerbated by multiple factors, including iron overload, lipid accumulation, peroxidation reactions, and transcription factor disorders. However, the significance of ferroptosis in neurodegenerative diseases and the proportion of nerve cell death it accounts for are critical questions. The heterogeneity of these diseases and the complex interplay of various cell death modalities have resulted in a lack of unified data on these issues, making them difficult to address.

In addition to the direct evidence provided by the research conclusions mentioned above, we believe that the correlations and contradictions among these findings deserve our in-depth consideration. For example, decreased expression of xCT following a disease affects the levels of GSH and GPX4. However, the cysteine deficiency caused by this decreased expression of xCT can also trigger autophagy, which in turn affects ferroptosis. Furthermore, iron overload may activate autophagy, and the bidirectional effects that arise after the activation of autophagy warrant further exploration.

Finally, considering the complex and intertwined mechanisms related to various cell death modalities, ferroptosis in neurodegenerative diseases may be studied through alternative approaches.

## Inhibiting Ferroptosis in Neurodegenerative Diseases

There are complex associations among aging, ferroptosis, and neurodegenerative diseases. On one hand, aging can increase susceptibility to ferroptosis and is also an important risk factor for neurodegenerative diseases. On the other hand, there is ample evidence supporting the role of ferroptosis in the occurrence and progression of neurodegenerative diseases, with the inhibition of ferroptosis believed to improve neurological function after the onset of these conditions (Wang et al., 2023c). Studies have shown that ferroptosis inhibitors, such as iron chelators and antioxidants, can exert neuroprotective effects (Yang et al., 2022; Wang et al., 2023e; Ayton et al., 2025). Additionally, the use of physical therapies, such as transcranial magnetic stimulation and stem cell-derived exosome therapy, represents a promising new direction for the treatment of neurodegenerative diseases.

### Ferroptosis inhibitors

Iron overload and oxidative stress in the nervous system are critical characteristics of neurodegenerative diseases. Therefore, long before ferroptosis was officially confirmed, a treatment plan for neurodegenerative diseases that involved iron ion chelation and antioxidation as therapeutic mechanisms was suggested and researched (Crapper McLachlan et al., 1991; Adair et al., 2001). Since the official discovery of ferroptosis, an increasing number of studies have shown its potential involvement in the pathogenesis of neurodegenerative diseases (Mi et al., 2019; Wang et al., 2023b; Currais et al., 2025). Consequently, research focused on improving neurological function through ferroptosis inhibitors has gained popularity among researchers (Gao et al., 2021; Hu et al., 2023; Yulug et al., 2023).

Due to the different mechanisms of ferroptosis in various neurodegenerative diseases, the targets and pharmacological mechanisms of related inhibitors also differ, with a particular emphasis on iron chelation and antioxidation. Given the limited space, only representative studies related to ferroptosis inhibitors in animal models and clinical studies of AD (**Additional Table 2**), PD (**Additional Table 3**), HD (**Additional Table 4**), and ALS (**Additional Table 5**) are discussed. While every piece of research deserves respect, we cannot subjectively judge right or wrong. However, by summarizing and analyzing the relevant studies, we can draw several conclusions. First, although the treatment of neurodegenerative diseases with ferroptosis inhibitors has been explored in many studies (Xie et al., 2018; Feng et al., 2021; Zhu et al., 2022), most of these investigations are limited to animal models, and few have been successfully translated into clinical studies (Cardoso et al., 2019; Ayton et al., 2025). AD and PD have been the focus of most existing clinical studies (Gao et al., 2021; Yu et al., 2023), and the sample sizes have generally been small. Furthermore, findings from previous clinical studies (Martin-Bastida et al., 2017; Mischley et al., 2017) suggest that the effects of ferroptosis inhibitors in neurodegenerative diseases are limited; some ferroptosis inhibitors cannot mitigate neurological dysfunction or cognitive impairment (Cardoso et al., 2019), and some may even exacerbate the disease (Ayton et al., 2025).

**Additional Table 2:** Research on ferroptosis inhibitors in AD

Target	Inhibitor	Experimental model	Mechanism	Outcome	Reference
Iron overload	DFO	SD rats	Iron chelator	Inhibit ferroptosis in AD model rat neurons	Zhu et al., 2022
	DFO	AD patients	Iron chelator	Slow the clinical progression of dementia	Crapper McLachlan et al., 1991
	Deferasirox	Albino rats	Iron chelator	Prevent iron accumulation and oxidative stress	Banerjee et al., 2016
	Clioquinol	Adult female ICR mice	Iron chelator	Improvements in cognition and memory	Li et al., 2022b
	DFP	AD patients	Iron chelator	Accelerate cognitive decline	Ayton et al., 2025
Lipid peroxidation	NDGA	Transgenic drosophila	Inhibit LOX	Reduce AD symptoms	Siddique and Ali, 2017
	CMS121	APPswe/PS1AE9- transgenic mice	Inhibit FASN	Alleviate memory decline	Ates et al., 2020
Antioxidant system disorder	Omega-3	Rats	Antioxidant	Protect brain microvascular endothelial cells	Wang et al., 2018
	Vitamin-E	Male Wistar rats	Antioxidant	Ameliorate memory and learning deficits	Mehrabadi and Sadr, 2020
	Vitamin-E	AD patients	Antioxidant	Cognitive status improved, unchanged or declined (based on heterogeneity)	Lloret et al., 2009
	Se	Triple transgenic AD model mice	Antioxidant	Ameliorate AD-related neuropathology and cognitive deficits	Xie et al., 2018
	Se	AD patients	Antioxidant	No significant beneficial effect on cognitive deficits	Cardoso et al., 2019
	FA	APP/PS1 mice	Activate Nrf2/GPX4 axis	Ameliorate AD-like symptoms	Wang et al., 2022a
	TSG	APP/PS1 mice	Activate GSH, GPX4 and RF2	Ameliorate memory and learning deficits	Gao et al., 2021
	Salidroside	Wild-type C57BL/6 mice	Activate Nrf2/HO1 axis	Mitigate thecognitive impairment induced by Aβ_1-42_	Yang et al., 2022
	ALA	AD patients	Antioxidant	Reduce cognitive deficits	Fava et al., 2013
	OAA	AD patients	Activate GSH	No significant amelioration of cognitive deficits	Vidoni et al., 2021
Other	PLs	Zebrafish	Modulate genes expression of ferroptosis	Improve swimming performance	Feng et al., 2021
	CMA	AD patients	Regulate critical proteins of AD	Improve cognitive function and clinical parameters associated with phenomics, metabolomics, proteomics and imaging analysis	Yulug et al., 2023

This table presents animal models studies and clinical trialsrelated to ferroptosis inhibitors of AD. AD: Alzheimer's disease; ALA: alpha-lipoic acid; CMA: combined metabolic activators; DFO: desferoxamine; DFP: deferiprone; FA: forsythoside A; FASN: fatty acid synthase; GPX4: glutathione peroxidase 4; GSH: glutathione; HO-1: heme oxygenase 1; LOX: lipoxygenase; PLs: plasmalogens; OAA: oxaloacetate; NDGA: nordihydroguaiaretic acid; NrF2: nuclear factor erythroid-2-related factor 2; SD: Sprague-Dawley; Se: selenium; TSG: tetrahydroxy stilbene glycoside.

**Additional Table 3:** Research on ferroptosis inhibitors in PD

Target	Inhibitor	Experimental model	Mechanism	Outcome	Reference
Iron overload	NBP	Adult male SD rats	Inhibition of iron deposition, oxidative stress, and ferroptosis	Alleviate motor disturbance	Hu et al., 2023
	DFP	PD patients	Iron chelator	Mitigate motor disturbance and improve quality of life, no statistical significance	Martin-Bastida et al., 2017
	DFP	PD patients	Iron chelator	Exacerbate motor and nonmotor symptoms	Devos et al., 2022
	DFP	PD patients	Iron chelator	Improve motor functions (early treatment compared with delayed treatment)	Devos et al., 2014
Lipid peroxidation	BHB	C57BL/6 mice	Modulate ACSL4 expression and antioxidative stress	Reduce oxidative stress and ferroptosis of dopaminergic neurons	Yu et al., 2023
Antioxidant	Fer-1	Wild-type zebrafish	Upregulate GPX4 expression	Inhibit ferroptosis in dopaminergic neurons	Sun et al., 2020
system disorder	Th A	Wild-type zebrafish	Activate Nrf2	Improve swimming performance	Sun et al., 2022a
	NAC	PD patients	Synthesize GSH	Improve dopaminergic function	Monti et al., 2019
	GSH	PD patients	Antioxidant	No significant beneficial effect compared with placebo	Mischley et al., 2017
	CoQ10	PD patients	Antioxidant	Improve ability to perform activities of daily living and motor function	Seet et al., 2014
	CoQ10	PD patients	Antioxidant	No significant clinical benefit	Parkinson Study Group QE3 Investigators et al., 2014
	Omega-3 and vitamin E	PD patients	Antioxidant	Ameliorate PD-like symptoms	Taghizadeh et al., 2017
	NAC	PD patients	Antioxidant and GSH synthesis	No significant decreases in oxidative damage	Coles et al., 2018
	GSH	PD patients	Antioxidant	No significant beneficial effect compared with placebo	Hauser et al., 2009
	Vitamin D	PD patients	Antioxidant	Reduce disease severity and anxiety	Zali et al., 2024
Mitochondrial	UDCA	PD patients	Improve mitochondrial function	Slightly beneficial effect compared with placebo, no	Payne et al., 2023
protection	NAD/NR	PD patients	Improve mitochondrial function	statistical significance Clinical improvement in motor symptoms	Berven et al., 2023
Other	Apoferritin	C57BL/6J male mice	Inhibit iron aggregation and regulate ACSL4 and FSP1	Mitigate motor deficits	Song et al., 2021
	Hinokitiol	Wild-type zebrafish	Iron chelation and modulation of the Nrf2 pathway	Rescue locomotor and neurodevelopmental deficits	Xi et al., 2022

This table presents animal models studies and clinical trials related to ferroptosis inhibitors of PD.ACSL4: Acyl-CoA synthetase long-chain family member 4; BHB: 3-hydroxybutyrate; DFP: deferiprone; Fer-1: ferrostatin-1; FSP1: ferroptosis suppressor protein 1; GPX4: glutathione peroxidase 4; GSH: glutathione; NAC: N-acetyl-cysteine; NAD: nicotinamide adenine dinucleotide; NBP: DL-3-N-butylphthalide; NR: nicotinamide riboside; NrF2: nuclear factor erythroid-2-related factor 2; PD: Parkinson's disease; SD: Sprague-Dawley; Th A: thonningianin A; UDCA: ursodeoxycholic acid.

**Additional Table 4:** Research on ferroptosis inhibitors in HD

Target	Inhibitor	Experimental model	Mechanism	Outcome	Reference
Iron overload	DFP	R6/2 and YAC128 mice	Iron chelator	Increase motor endurance	Agrawal et al., 2018
Lipid peroxidation	D-Ala2GIP	Male Wistar rats	Attenuate lipid peroxidation	Attenuate the neurobehavioral deficits	Verma et al., 2018
	D-PUFA	Q140 mice	Inhibit lipid peroxidation	Significant improvement in cognitive performance	Hatami et al., 2018
Antioxidant system	XJB-5-131	R6/2 mice	Antioxidant	Ameliorate motor and temperature regulation deficits	Polyzos et al., 2018
disorder	CoQ10	HD patients	Antioxidant	No significant clinical benefit	McGarry et al., 2017
	Vitamin D3	Male C57BL/6 mice	Antioxidant and anti-inflammatory	Rescue deficits in cholinergic neurotransmission	Manjari et al., 2023
	Se	Male C57BL/6J mice	Antioxidant and anti-inflammatory	Ameliorate cognitive deficits	Liang et al., 2023b
	Cysteamine	HD patients	Antioxidant	Slight reduction in deterioration, no statistical significance	Verny et al., 2017
Mitochondrial protection	Laduviglusib	Postmortem samples of HD patients	Inhibit ferroptosis to improve function of mitochondria in the striatum	Neuroprotective effects	Liu et al., 2024a
	Quercetin	Female Wistar rats	Antioxidant and ameliorate mitochondrial dysfunction	Attenuate the neurobehavioral deficits	Sandhir and Mehrotra, 2013
	NAC	R6/1 transgenic mouse	Ameliorate mitochondrial dysfunction	Delay the onset of motor deficits	Wright et al., 2015
	EPA	HD patients	Stabilize mitochondrial integrity	No significant clinical beneficial effect on motor function	Ferreira et al., 2015

This table presents animal models studies and clinical trials related to ferroptosis inhibitors of HD. DFP: Deferiprone; D-PUFA: deuterated polyunsaturated fatty acids; EPA: ethyl- eicosapentaenoic acid; HD: Huntington's disease; NAC: N-acetyl-cysteine; Se: selenium.

**Additional Table 5:** Research on ferroptosis inhibitors in ALS

Target	Inhibitor	Experimental model	Mechanism	Outcome	Reference
Iron overload	DFO	SOD1(G93A) mice	Iron chelator	Delay disease onset and extend lifespan	Lee et al., 2015
	Compound M30	SOD1(G93A) mice	Iron chelator	Delay the onset of motor dysfunction and extend lifespan	Kupershmidt and Youdim, 2023
	DFP	SOD186R transgenic mice and ALS patients	Iron chelator	Extend lifespan in mice, slow disease progression and reduce weight loss in ALS patients	Moreau et al., 2018
Lipid peroxidation	RT001	ALS patients	Inhibit lipid peroxidation and improve mitochondrial function	Slow disease progression	Weemering et al., 2023
Antioxidant system	Edaravone	SOD1(G93A) mice	Antioxidant	Alleviate the degeneration of motor neurons and muscles	Ohta et al., 2019
disorder	Edaravone	ALS patients	Antioxidant	Slow disease progression	Nagase et al., 2016
	Edaravone	ALS patients	Antioxidant	No significant clinical benefit	Witzel et al., 2022
	Edaravone	ALS patients	Antioxidant	Slow disease progression in some patients; ineffective for the majority of patients	Writing Group and Edaravone MCI-186 ALS 19 Study Group, 2017
	Riluzole	ALS patients	Glutamatergic antagonist	Prolong survival of patients with ALS in last clinical stage	Fang et al., 2018
	Nanocurcumin	ALS patients	Antioxidant	Increase the probability of survival as add-on therapy to riluzole	Ahmadi et al., 2018
	Methylcobalamin	ALS patients	Antioxidant and anti-inflammatory	Efficacious in slowing functional decline in patients with early-stage ALS and with moderate progression rate	Oki et al., 2022
	HNK	SOD1(G93A) mice	Antioxidant and ameliorate mitochondrial dysfunction	Extend the lifespan and improve the motor function	Zhou et al., 2023
	Urate	Transgenic Drosophila stocks	Antioxidant and synthesis GSH	Increase survival and strengthen motor activity	Zhang et al., 2019
	PUFAs and vitamin E	ALS patients	Antioxidant	Reduce the risk of developing ALS	Veldink et al., 2007
	CoQ10	ALS patients	Antioxidant and ameliorate mitochondrial dysfunction	No significant clinical benefit	Kaufmann et al., 2009
Other	Sodium phenylbutyrate-taurursodiol	ALS patients	Mitigate endoplasmic reticulum stress and mitochondrial dysfunction	Slow disease progression, no statistical significance	Paganoni et al., 2020

This table presents animal models studies and clinical trials related to ferroptosis inhibitors of ALS. ALS: Amyotrophic lateral sclerosis; Compound M30: 5-[N-methyl-N- propargylaminomethyl]-8-hydroxyquinoline; DFP: deferiprone; DFO: desferoxamine; HNK: honokiol; GSH: glutathione; PUFA: polyunsaturated fatty acid; SOD1: superoxide dismutase 1.

Finally, the existing mechanisms of action of ferroptosis inhibitors in neurodegenerative diseases are relatively singular, primarily involving iron chelators or antioxidants. Researchers should explore other mechanistic targets of ferroptosis, such as those involved in autophagy, lipid metabolism, and epigenetic modulation. While further studies involving other mechanisms or targets may be in progress, they may not be directly aimed at regulating ferroptosis. The limitations mentioned above may be partly attributed to bias arising from small sample sizes (Cardoso et al., 2019), excessive dropout rates (Lloret et al., 2009), and overly high expectations for therapeutic effects (Kaufmann et al., 2009).

After an in-depth analysis of the 33 clinical studies on ferroptosis inhibitors presented in the table above, we found that 15 of them clearly confirmed that ferroptosis inhibitors could significantly improve clinical outcomes for patients, accounting for 45.5%. The mechanisms involved included iron chelation (Crapper McLachlan et al., 1991), antioxidation (Seet et al., 2014), improved mitochondrial function (Berven et al., 2023), and anti-inflammatory effects (Oki et al., 2022). Additionally, another six studies (18.2%) suggested that ferroptosis inhibitors have the potential or trend to improve patient prognosis. However, the conclusions of the remaining 12 studies (36.4%) did not provide positive evidence for the efficacy of ferroptosis inhibitors, with 2 studies (6.1%) reporting that these inhibitors aggravated the disease. A specific analysis of each disease revealed the following results: Out of seven studies, three demonstrated clear therapeutic effects, one showed improved prognosis, two reported no improvement, and one indicated disease aggravation. Among thirteen studies, five had definite therapeutic effects, three showed improved prognosis, four reported no improvement, and one indicated disease aggravation. Of three studies, one showed improved prognosis, while two reported no improvement. Out of ten studies, seven showed definite therapeutic effects, one indicated improved prognosis, and two reported no improvement. A specific analysis of specific ferroptosis inhibitors showed that four studies involving DFP were conducted, with two showing potential efficacy but lacking definitive evidence or statistical significance, and two accelerating disease deterioration. A total of four studies invovling CoQ10 were reviewed, with one showing improved prognosis and three showing no difference. Of the three studies involving vitamin E, two demonstrated clear efficacy, while one showed varying degrees of efficacy depending on patient heterogeneity. Of three studies involving Edaravone, two showed definite efficacy, and one did not confirm the efficacy of short-term treatment. The results of two studies regarding GSH did not support a clear improvement in prognosis.

Most of the studies that failed to demonstrate the efficacy of ferroptosis inhibitors provided corresponding explanations, including but not limited to the following: the “placebo effect” of certain behaviors during treatment (Mischley et al., 2017), the inability of drugs to penetrate the blood–brain barrier (Hauser et al., 2009), low oral drug absorption (Coles et al., 2018), insufficient treatment duration (Witzel et al., 2022), small sample sizes (Hauser et al., 2009), outdated or inadequate detection methods (McGarry et al., 2017), and the absence of a control group (Vidoni et al., 2021). Additionally, we would like to highlight two studies that suggest ferroptosis inhibitors, specifically the iron chelator DFP, may accelerate disease progression. The reasons for this include: DFP reduces the activity of tyrosine hydroxylase, thereby affecting dopamine synthesis (Devos et al., 2022), and DFP accelerates the loss of hippocampal volume (Ayton et al., 2025).

In addition, we found contradictions between the conclusions of some clinical studies. After comparing these studies with conflicting conclusions, we summarized several reasons. First, due to individual differences in the population, known as heterogeneity, vitamin E exhibits varying sensitivities in different patients, which in turn affects its overall efficacy (Lloret et al., 2009). Second, the differences in the grouping of patients participating in the experiments also influenced the conclusions. One study suggested that early treatment with DFP was more effective than delayed treatment; however, it failed to establish a control group, making it impossible to confirm whether the treatment was truly effective (Devos et al., 2014). Additionally, differences in treatment duration affect the final results. CoQ10 was applied to improve neurological function after PD, and an improvement in scores was observed after 8 weeks of short-term treatment (Seet et al., 2014). However, no clear improvement was noted after 16 months of long-term treatment (Parkinson Study Group QE3 Investigators et al., 2014). Moreover, different dosages and administration methods can impact efficacy. Symptom improvement was observed with intravenous NAC for PD, while no significant difference was found with oral administration (Coles et al., 2018). Finally, differences in sample size can also lead to bias in the results.

Edaravone demonstrated a definite ability to delay the progression of ALS in small sample studies (26 cases and 137 cases) (Nagase et al., 2016; Writing Group and Edaravone (MCI-186) ALS 19 Study Group, 2017). However, in a larger sample study (324 cases), additional edaravone treatment failed to show significant differences compared with conventional treatment with riluzole (Witzel et al., 2022). It is worth noting that in the aforementioned studies on edaravone, variations in dosage and duration also impact the final results. Lastly, different stages of the disease contribute to contradictions in the research findings. Riluzole has been shown to significantly prolong the survival of patients with advanced (stage 4) ALS, but it has no significant effect on patients with intermediate (stages 2 and 3) ALS (Fang et al., 2018).

In addition to efficacy, the safety of drugs is also an important indicator to consider in clinical trials. The safety of all ferroptosis inhibitors involved in the studies mentioned above was generally excellent, although the safety of drugs used in a few studies was unsatisfactory (Paganoni et al., 2020). In general, the vast majority of patients tolerate these drugs well, with only a few experiencing sporadic adverse events. Few serious adverse events have been reported, and such events are rarely directly related to the drugs. Adverse events associated with drug safety mainly include gastrointestinal symptoms following medication (Oki et al., 2022; Payne et al., 2023) and injuries related to intravenous medication (Witzel et al., 2022). It is worth noting that the types and frequency of adverse events associated with different drugs vary. Iron chelators, such as DFP, are primarily associated with neutropenia (Devos et al., 2014, 2022). Coenzymes, represented by vitamins and CoQ10, mainly result in gastrointestinal symptoms (McGarry et al., 2017). However, edaravone has been shown to cause drug allergies and injuries related to intravenous medication (Witzel et al., 2022).

### Physical therapy

Through a comprehensive review of the relevant literature, we believe that physical therapies, represented by noninvasive brain stimulation, electroacupuncture, electrical stimulation (ES), and photobiomodulation, can improve patient prognosis by inhibiting ferroptosis in neurodegenerative diseases. Although there is a lack of direct evidence, numerous studies have confirmed the role of physical therapies in combating oxidative stress (Medina-Fernández et al., 2018; Jiang et al., 2023; Uemura et al., 2023). Therefore, we hypothesize that physical therapies inhibit ferroptosis through their anti-oxidative stress effects.

#### Transcranial magnetic stimulation

Noninvasive brain stimulation mainly includes transcranial magnetic stimulation (TMS) and transcranial direct current stimulation (tDCS). Clinical studies related to neurodegenerative diseases, including AD (Jung et al., 2024), PD (Ji et al., 2021; Grobe-Einsler et al., 2024), HD (Brusa et al., 2005), and ALS (Zanette et al., 2008), have demonstrated remarkable ability of TMS to mitigate patients’ neurological dysfunction and cognitive impairment. Research has shown that TMS possesses antioxidant capacity (Medina-Fernández et al., 2018). Cao et al. (2022) reported that high-frequency repetitive TMS (rTMS) at 25 Hz effectively reduced oxidative stress levels in AD model mice by regulating the PI3K/AKT/glutamate transporter 1 axis, thereby alleviating cognitive impairment. It is well known that high levels of glutamate can induce ferroptosis (Yang et al., 2022). TMS is believed to regulate glutamate concentration; for example, Poh et al. (2019) demonstrated that rTMS reduces Glu levels in the nervous system. Additionally, rTMS promotes the expression of glutamate transporter genes and reduces glutamate accumulation in neurons (Ikeda et al., 2019). Unfortunately, there is no relevant direct research on the ability of tDCS to inhibit ferroptosis.

#### Electroacupuncture

Previous studies have shown that EA can downregulate ferroptosis by significantly reducing oxidative stress, inhibiting iron overload, and activating antioxidant pathways (Wan et al., 2023; Xue et al., 2024). Jiang et al. (2023) reported that EA alleviated cerebrovascular damage in AD model mice by driving melatonin signaling, while melatonin helped alleviate cognitive deficits by regulating mitophagy flux, neuroinflammation, and oxidative stress. Additionally, EA decreases glutamate levels and reduces the levels of glutamate neurotransmission-related proteins in the hippocampus in a post-traumatic stress disorder model of AD, thereby lowering the risk of glutamate-induced ferroptosis (Cai et al., 2023). In a PD rat model, EA effectively decreased oxidative stress and lipid peroxidation markers in the substantia nigra pars compacta, helping to prevent ferroptosis (Hu et al., 2024). Peng et al. (2017) investigated the effect of EA combined with donepezil to alleviate cognitive impairment in patients with AD; however, this study was not carried out for various reasons.

#### Electrical stimulation

As a noninvasive treatment, ES can play an antioxidant role by reducing cellular ROS generation and upregulating key components of the cellular antioxidant system, such as NADPH and α-ketoglutarate (Uemura et al., 2023). Findings from a rat model study by Chen et al. (2024a) confirmed that ES can significantly attenuate oxidative stress and ROS concentrations while increasing GSH concentrations. Degradation of iron deposits and protein aggregates was observed in AD model mice treated with an alternative electric field (Choi et al., 2023). Concurrently, a previous study has shown that ES can improve episodic memory and ameliorate cholinergic dysfunction in patients with AD (Benussi et al., 2022). A constant current electrical stimulator can effectively inhibit tremors in patients with PD (Gong et al., 2024). Unfortunately, we did not find direct evidence that ES ameliorated symptoms of neurodegenerative diseases by inhibiting ferroptosis.

#### Photobiomodulation

Photobiomodulation (PBM) has also been shown to have antioxidant effects (Kim and Won, 2022), thereby reducing the likelihood of ferroptosis induced by peroxidation, although direct supporting evidence is lacking. Additionally, brain PBM treatment is believed to increase neuronal metabolism, exert anti-inflammatory and antioxidant effects, and stimulate nerve and synapse regeneration (Salehpour et al., 2018). In animal and cellular models, PBM can ameliorate cognitive impairment following the development of AD (Enengl et al., 2020). A subsequent study by Su et al. (2023) demonstrated that PBM inhibits oxidative stress in AD, reducing Aβ plaque formation and thereby delaying the onset and progression of the disease. In a double-blind, randomized, sham-controlled feasibility trial by Herkes et al. (2023), transcranial PBM was shown to be a safe and feasible non-drug adjuvant therapy for PD, with one of its possible mechanisms of action being the inhibition of oxidative stress to improve PD motor symptoms.

### Stem cells and exosomes

#### Stem cells therapy

Stem cells, especially induced pluripotent stem cells, are increasingly becoming a promising option for the treatment of neurodegenerative diseases (Giacomelli et al., 2022; Wang et al., 2022e). Meanwhile, findings from a growing number of studies have confirmed that stem cells can effectively inhibit ferroptosis (Feng et al., 2022; Yao et al., 2023). For example, Yao et al. (2023) used mesenchymal stem cells (MSCs) to target mitochondrial quality control to rescue neuronal mitochondrial homeostasis, thereby downregulating neuronal ferroptosis. Feng et al. (2022) reported that human umbilical cord-derived MSCs (HUC-MSCs) can effectively reduce oxidative stress levels in smooth muscle and upregulate the expression of antioxidant factors such as GPX4 and SLC7A11. However, studies on stem cells targeting ferroptosis have mostly focused on other diseases (Feng et al., 2022; Yao et al., 2023), with almost no direct studies on neurodegenerative diseases. Wang et al. (2022b) reported that the deposition of pathological proteins such as Aβ and tau decreased after the administration of MSCs to traumatic brain injury model mice. At the same time, iron accumulation was inhibited, antioxidant capacity was restored, levels of lipid peroxidation markers were reduced, ferroptosis was downregulated, and cognition was improved (Wang et al., 2022b). Notably, this study confirmed that the effects of MSCs after administration were similar to those of ferroptosis inhibitors.

#### Exosomes therapy

Stem cell therapy has great potential, but risks such as tumorigenicity, immunogenicity, and heterogeneity are concerning (Yamanaka, 2020). Additionally, ethical conflicts (Hyun, 2022), rapid aging during *in vitro* culture and expansion (Yuan et al., 2020), must also be addressed. With in-depth research on stem cell-derived exosomes, researchers have demonstrated that exosomes possess nearly all the functions of stem cells. Furthermore, exosomes are safer and more reliable than stem cells (Tan et al., 2024). There is no doubt that exosomes also inhibit ferroptosis (Yang et al., 2024b). Adipose-derived mesenchymal stem cell exosomes can downregulate the expression of ferroptosis-related proteins and promote the recovery of neurological function in mice (Wang et al., 2023d). Exosomes from HUC-MSCs activate mitophagy through the PTEN-induced putative kinase 1/Parkin RBR E3 ubiquitin-protein ligase (PINK1/PARKIN) pathway, thereby inhibiting ferroptosis and exerting neuroprotective functions (Zhang et al., 2023). HUC-MSCs-derived exosomes have also been shown to inhibit ferroptosis after nerve injury by activating antioxidant pathways (Yang et al., 2024a). In addition to their direct roles, exosomes can also act as drug carriers. For example, Wang et al. (2024a) used brain-derived neurotrophic factor-loaded HUC-MSCs-derived exosomes to intervene in animal and cell models of PD and observed significant antioxidant effects and downregulation of ferroptosis. Moreover, compared with stem cell administration, exosome administration is more convenient, with various methods of administration available, including intranasal delivery (Wang et al., 2023d) and conventional injection (Yang et al., 2024a).

### Future therapy prospects

As we have mentioned multiple times above, the regulation of ferroptosis by autophagy is bidirectional, depending on the specific conditions. Therefore, the ultimate goal of inhibiting ferroptosis can be achieved through the regulation of autophagy. Ferritinophagy is a crucial factor that triggers ferroptosis, and NCOA4 is considered to be central to the mechanisms related to ferritinophagy. Some studies have confirmed the feasibility of inhibiting ferroptosis by regulating NCOA4 to downregulate ferritinophagy (An et al., 2024; Sun et al., 2024b). The regulation of ferroptosis by transcription factors is also bidirectional, allowing for the negative regulation of ferroptosis through interference. Previous studies have confirmed that transcription factors participate in ferroptosis in neurodegenerative diseases and that their ability to regulate transcription can inhibit ferroptosis, thereby reduce cognitive impairment and neurological dysfunction (Lv et al., 2024; Deng et al., 2025). Additionally, it is theoretically feasible to intervene in iron metabolism and oxidation reactions by regulating the microenvironment. Furthermore, some natural compounds, especially polyphenols, exhibit significant properties as inhibitors of ferroptosis (Russo et al., 2025). Finally, supplementing the raw materials required for the synthesis of the antioxidant system also appears to be a viable option.

However, given that the significance of ferroptosis in neurodegenerative diseases is not yet fully understood, we must acknowledge that there are doubts about whether therapies aimed at inhibiting ferroptosis are definitive. Additionally, therapies aimed at inhibiting ferroptosis may serve other purposes, but we have not yet achieved those objectives.

## Limitations

The existing studies on ferroptosis in neurodegenerative diseases still have many limitations (Mischley et al., 2017; Ayton et al., 2025). First, while our understanding of the mechanisms of ferroptosis has increased, it is important to note that, in addition to iron overload, lipid peroxidation, and antioxidant system imbalances, factors such as imbalances in cell membrane repair, autophagy, and gene regulation have also been shown to participate in ferroptosis. Research on ferroptosis in neurodegenerative diseases has primarily focused on iron accumulation and lipid peroxidation, with less attention given to other processes. Second, most relevant studies have addressed on AD and PD, with fewer studies focused on HD and ALS, likely due to the lower incidence of these latter diseases. Furthermore, studies on interventions targeting ferroptosis in neurodegenerative diseases have predominantly examined ferroptosis inhibitors, with most being animal or cell studies and limited clinical translation. The few existing clinical cohort studies also face challenges such as small sample sizes and low completion rates. Additionally, some research findings have suggested that the efficacy of ferroptosis inhibitors is not ideal. Furthermore, physical therapies, stem cell treatments, and exosome therapies may inhibit ferroptosis. The mechanisms of these therapies are diverse, their scope of action is broad, and there is indirect evidence supporting their effectiveness; however, direct research in this area is still lacking. Finally, given that the significance of ferroptosis in neurodegenerative diseases remains unclear, we cannot determine whether inhibiting ferroptosis in these conditions can achieve a definitive effect.

In addition, this narrative review has certain limitations. On one hand, due to our academic level, we have presented specific viewpoints in the article, but we still lack a more in-depth exploration of the existing research findings. On the other hand, in this review, we primarily focus on direct studies related to the main theme and rarely address related indirect studies.

## Conclusions

With the aging population, the incidence of age-related neurodegenerative diseases, such as AD, PD, HD, and ALS, has increased annually, resulting in disastrous consequences. As individuals age, iron and lipid accumulation, imbalances in the antioxidant system, and autophagy disorders inevitably occur in the human nervous system. These phenomena can increase susceptibility to ferroptosis. Findings from recent studies have confirmed that ferroptosis is involved in age-related neurodegenerative diseases and may be one of the pathogenic mechanisms underlying these disorders. Recent research has shown that inhibiting ferroptosis can effectively mitigate the neurological dysfunction and cognitive impairment associated with neurodegenerative diseases. The ability of ferroptosis inhibitors, as well as physical therapies and stem cell and exosome therapies, to inhibit ferroptosis has been demonstrated.

In summary, this review has summarized progress in research on the mechanisms of ferroptosis, the associations between ferroptosis and aging, the relationships between ferroptosis and neurodegenerative diseases, and related intervention therapies. Aging is a common risk factor for both ferroptosis and neurodegenerative diseases, and ferroptosis may be an important pathogenic mechanism underlying these conditions. Inhibiting ferroptosis is expected to effectively mitigate neurological dysfunction and cognitive impairment in patients with neurodegenerative diseases. Although neurodegenerative diseases remain incurable and the existing research conclusions are not yet satisfactory, the available results at least point to a new direction. We believe that with in-depth research, we will be closer to achieving the ultimate goal of overcoming neurodegenerative diseases.

## Additional files:

***Additional Table 1:*** *Various forms of cell death.*

***Additional Table 2:*** *Research on ferroptosis inhibitors in AD.*

***Additional Table 3:*** *Research on ferroptosis inhibitors in PD.*

***Additional Table 4:*** *Research on ferroptosis inhibitors in HD.*

***Additional Table 5:*** *Research on ferroptosis inhibitors in ALS.*

## Data Availability

*All relevant data are within the paper and its Additional files*.
